# Solid Electrolyte Interphase Formation in Tellurium Iodide Perovskites during Electrochemistry and Photoelectrochemistry

**DOI:** 10.1021/acsami.3c07425

**Published:** 2023-07-24

**Authors:** Yuhan Liu, Yuting Yao, Xinyue Zhang, Christopher Blackman, Robin S. Perry, Robert G. Palgrave

**Affiliations:** † Department of Chemistry, University College London, Christopher Ingold Building, 20 Gordon Street, London WC1H 0AJ, U.K.; ‡ London Centre for Nanotechnology and Department of Physics and Astronomy, University College London, 17-19 Gordon Street, London WC1H 0AH, U.K.; § ISIS Neutron Spallation Source, Rutherford Appleton Laboratory, Harwell Campus, Didcot OX11 0QX, UK

**Keywords:** halide perovskites, photoelectrochemistry, interface, tellurium, solid electrolyte interphase

## Abstract

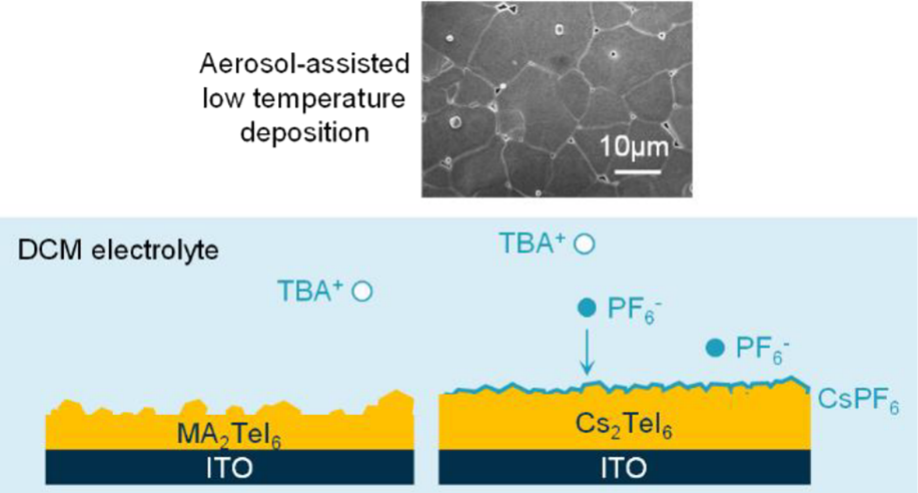

Halide perovskites are promising photoelectrocatalytic materials. Their further development requires understanding of surface processes during electrochemistry. Thin films of tellurium-based vacancy-ordered perovskites with formula A_2_TeI_6_, A = Cs, methylammonium (MA), were deposited onto transparent conducting substrates using aerosol-assisted chemical vapor deposition. Thin film stability as electrodes and photoelectrodes was tested in dichloromethane containing tetrabutylammonium PF_6_ (TBAPF_6_). Using photoemission spectroscopy, we show that the formation of a solid electrolyte interphase on the surface of the Cs_2_TeI_6_, consisting of CsPF_6_, enhances the stability of the electrode and allows extended chopped-light chronoamperometry measurements at up to 1.1 V with a photocurrent density of 16 μA/cm^2^. In contrast, (CH_3_NH_3_)_2_TeI_6_ does not form a passivating layer and rapidly degrades upon identical electrochemical treatment. This demonstrates the importance of surface chemistry in halide perovskite electrochemistry and photoelectrocatalysis.

## Introduction

Hybrid lead and tin iodide perovskites display many ideal properties for photovoltaic and photoelectrochemical applications; however, poor long-term stability and toxicity have directed some attention to other lead-free perovskite and perovskite-related materials.^1−4^ Inorganic and hybrid tellurium halides (A_2_TeX_6_, X = Br, I, A = Cs, MA) are a possible alternative having the vacancy-ordered cubic structure *Fm*3̅*m*, with an ideal band gap and good stability.^5,6^ Furthermore, Te^4+^ has a similar electronic structure to Sn^2+^ (5s^2^ electronic configuration) but significantly greater stability, making it a promising substitute.^7−9^

An increasingly attractive application for halide perovskites beyond photovoltaics is electrochemistry (EC) and photoelectrochemistry (PEC), where properties such as long carrier diffusion, tunable band edge positions, and band gap should enable design of new, effective electrode materials, which can be used for solar energy capture or for driving chemical transformations.^10−14^ EC and PEC reactions have been carried out in aqueous and nonaqueous electrolytes using perovskites such as CsPbBr_3_ and MAPbI_3_,^13^ Cs_2_PtBr_6_,^15^ and Cs_2_PtI_6_.^16,17^ CsPbBr_3_ nanoparticles showed promise for electrochemical conversion of organic molecules,^18^ while Cs_2_PtI_6_ formed useful heterojunctions with BiVO_4_ allowing photogenerated charge separation.^19^ A common difficulty that is present in all these materials when used for EC or PEC is surface stability, both on initial contact with the electrolyte and under illumination and applied or photogenerated bias. Jayaraman et al. found that Cs_2_PtI_6_ was the most stable of the perovskite materials they studied.^17^ Pornrungroj et al. employed a graphite epoxy encapsulation method to improve the stability of their lead-based perovskite photocathodes;^19^ metal alloy layers have also been used for passivation.^20,21^ However, while a lot of attention has focused on stabilizing perovskite surfaces for electrochemistry, little work has been done to understand what chemical processes lead to degradation, and whether design of materials might be able to prevent this. Here, we present a surface study on A_2_TeI_6_ electrodes in a nonaqueous PEC reaction. We show that all-inorganic Cs_2_TeI_6_ films form a passivation layer during initial cycling in the electrolyte through reaction with solution-phase tetrabutylammonium PF_6_ (TBAPF_6_). This layer operates as a solid electrolyte interphase (SEI) and stabilizes the film, preventing bulk degradation, while allowing extended chronoamperometry to occur. However, the hybrid analogue MA_2_TeI_6_ cannot form such a SEI layer and is rapidly degraded during electrochemical cycling. Our SEI layers thus fulfill a similar role to those formed on intercalation battery electrodes.

Perovskite film thickness, crystallinity, and morphology determine the device performance, and the fabrication process determines these properties.^22,23^ A highly crystalline, dense morphology, and pin-hole free thin film is required for a low recombination rate of photoelectrons and hence a high device efficiency,^24^ and this design principle is expected to hold for PEC devices as well as solar cells. PEC electrodes are made by either deposition of a colloidal solution of perovskite nanoparticles, or deposition of a flat film using spin coating; however, this is difficult to scale up due to substrate size limitations. Other techniques such as vapor deposition may be limited by high-vacuum or high-cost, impeding mass production.^22^

Aerosol-assisted chemical vapor deposition (AACVD) is a simple, low-cost, ambient pressure technique that can be applied to large-scale deposition. A nebulizer is used to generate a precursor mist which is then transported into the AACVD chamber by a carrier gas. The solvent is evaporated at the substrate driving the deposition inside the chamber. In 2014, one-step deposition of the CH_3_NH_3_PbBr_3_ thin film using AACVD was first reported by Lewis and O’Brien.^25^ In 2015, CH_3_NH_3_PbI_3_ deposition was achieved using AACVD at 200 °C,^26^ although the coating was low-density and unsuitable for applications indicating that further work was required;^27^ a low-temperature deposition was achieved by ultrasonic spraying coating;^28^ high-quality films were obtained at 75 °C using a DMF solvent, which prevented desiccation of the film and generated a pin-hole-free surface. In 2018, Cs_2_SnI_6_ film deposition was achieved on ITO substrates via AACVD at 130 °C.^29^

Previously, MA_2_TeI_6_ thin film synthesis has been attempted in a number of ways: spin-coated films were poor quality while synthesis by a thermal evaporation process was promising.^5^ Furthermore, electrospray in air has been applied to grow Cs_2_TeI_6_ thick films^7,30^ and later, one-step spin coating of Cs_2_TeI_6_ films was investigated by Vázquez-Fernández et al.^9^ Here, we demonstrate a low-temperature aerosol-assisted deposition process, which uses a similar setup to AACVD, to deposit highly preferentially oriented, high-density cubic structure Te-based perovskite thin films. The electrochemical and PEC performances are studied with the as-synthesized Cs_2_TeI_6_ and MA_2_TeI_6_ thin films with the assistance of photoelectron spectroscopy.

## Experimental Procedures

### Materials

Cs_2_CO_3_ (99%), TeO_2_ (≥99%), methylamine solution (MA, 40 wt % in H_2_O), hydriodic acid (HI, 57% wt % in H_2_O), dimethyl sulfoxide (DMSO, anhydrous, ≥99.9%), and tetrabutylammonium hexafluorophosphate (TBAPF_6_) were purchased from Sigma-Aldrich; hydrobromic acid (HBr, 47–49% wt % in H_2_O) was purchased from Alfa Aesar and *N*,*N*-dimethylformamide (DMF) was purchased from SERVA. Dichloromethane (DCM, ≥99.8%), diethyl ether (≥99.5%), and acetonitrile (≥99.8%) were purchased from Fisher Scientific. ITO substrates were purchased from Luoyang GULUO Glass Co., LTD. All the materials (except ITO) and solvents were used as received.

### Synthesis of Perovskites

#### MA_2_TeI_6_

5 mmol (0.777 g) MA solution was added into 10 mL of HI and stirred for 10 min to obtain a clear solution; 5 mmol (0.798 g) TeO_2_ and 15 mL of HI were added and stirred for 30 min in 100 mL of acetonitrile in which the product is found to have a higher solubility than water. MAI/HI solution was quickly added into TeI_4_/HI/acetonitrile solution, and the mixture was stirred for another 10 min at 80 °C to evaporate acetonitrile and precipitate the product. The final product MA_2_TeI_6_ was then vacuum-filtered, washed with diethyl ether, and dried at 60 °C.

#### Cs_2_TeI_6_

5 mmol (1.629 g) Cs_2_CO_3_ was added into 10 mL of HI and stirred for 10 min to obtain a clear solution; 5 mmol (0.798 g) TeO_2_ and 15 mL of HI were added and stirred for 30 min in 100 mL of acetonitrile. CsI/HI solution was quickly added into TeI_4_/HI/acetonitrile solution, and the mixture was stirred for another 10 min at room temperature. The final product Cs_2_TeI_6_ was vacuum-filtered, washed with diethyl ether, and dried at 60 °C.

### Electrochemical Characterization

All the electrochemical studies were carried out with a three-electrode cell setup using a GAMRY workstation. Ag/AgCl (3 M KCl) and Pt coil were used as the reference electrode and counter electrode, respectively. The electrolyte and supporting electrolyte were DCM and 0.01 M TBAPF_6_, respectively. Cyclic voltammetry (CV) measurements were carried out between −1.5 and 2.5 V (vs Ag/AgCl) at a scan rate of 5 mV/s.

Electrochemical impedance spectroscopy (EIS) measurements were carried out with the frequency range from 10 Hz to 50 mHz, voltages from 1.0 to −0.8 V (vs Ag/AgCl) with a voltage interval of 0.1 V, superimposed with a sinusoidal AC amplitude of 10 mV. The *Z*_imaginary_ data were equated into the capacitance with the formula *Z*_im_ = 1/ω*C*. Mott–Schottky plots of 1/*C*^2^ vs potential were obtained at 2.254, 1.002, and 0.3167 Hz.

### PEC Characterization

PEC measurements were performed under simulated AM1.5G sunlight using a xenon lamp. Linear sweep voltammetry (LSV) was carried out between 2.5 and −1.5 V (vs Ag/AgCl) at a scan rate of 2 mV/s. Chopped-light measurements were done using a homemade chopper positioned between the light source and the PEC cell; the on/off time was 10/10 s. Open circuit potentials (OCPs) were measured in the dark and under different irradiance to extract the flat band potential.

### Powder X-Ray Diffraction (PXRD)

PXRD data were obtained by a Stoe Stadi-P X-ray diffractometer with a Mo Kα_1_ (λ = 0.70930 Å) radiation source operated at 50 kV, 30 mA. All patterns were obtained by using a step scan method (0.5° per step for 10 s) in transmission mode, in a 2θ range from 2° to 40°.

### Grazing-Incidence XRD (GIXRD)

GIXRD data were collected by a Bruker D8 X-ray diffractometer. The radiation source was Cu Kα (λ = 1.5406 Å) operated at 20 kV, 5 mA. All patterns were obtained by using a step scan method (0.05° per step for 2 s), in a 2θ range from 10° to 50°.

### Scanning Electron Microscopy (SEM) with Energy-Dispersive Spectroscopy (EDS)

A Jeol JSM-7600 Scanning Electron Microscope was used to observe the morphology of films. Sputter coating of gold on the surface of the film before the SEM process to obtain a good conductivity of film thus a better quality of images. Gold nanoparticles are found difficult to spread on the surface of perovskites due to unknown reasons, which leads to the bright spots in SEM images. The mapping for elemental distribution and composition was acquired by SEM-incorporated EDS with an accelerating voltage of 15 keV; data were collected by AZtecOne from Oxford Instruments.

### UV–Vis Measurement

A UV-2600 spectrophotometer (SHIMADZU) was used for UV–vis measurement. Optical absorbance *A*(λ) and reflectance *R*(λ) were measured in the spectral wavelength range of 200–900 nm. Tauc plots were obtained from absorption spectra transformed from reflectance by the Kubelka–Munk equation, and then the optical bandgap can be calculated based on the Tauc plot.^31,32^ The absorption coefficient was calculated from absorbance spectra, and the thin film thickness was measured with SEM. Electrolyte absorption spectra were measured using 0.01 M TBAPF_6_ in DCM as the reference to observe the dissolved ions after redox reactions.

### X-Ray Photoelectron Spectroscopy (XPS)

XPS was measured by a Thermo K-alpha spectrometer, which uses an Al Kα X-ray source (*h*ν = 1486.6 eV, calibrated by the adventitious carbon 1s peak (285 eV) or I^–^ 3d_5/2_ peak (618.7 eV). The diameter of the X-ray beam was set as 400 microns on the surface. A dual-beam flood gun was used for charge compensation. Data were collected at 200 eV pass energy for survey and 50 eV pass energy for core-level spectra. Radiation damage was observed on tellurium spectra as shown in Figure S13; therefore, an area scan was set up for tellurium and only one scan was taken on each point to avoid the beam damage as much as possible. All the other elements were scanned 20 times at 1 point. CasaXPS^33^ is used to analyze the valency states from XPS spectra and to calculate the composition from the peak area.

## Results and Discussion

### Low-Temperature Aerosol-Assisted Deposition of Halide Perovskites

**Figure 1:**
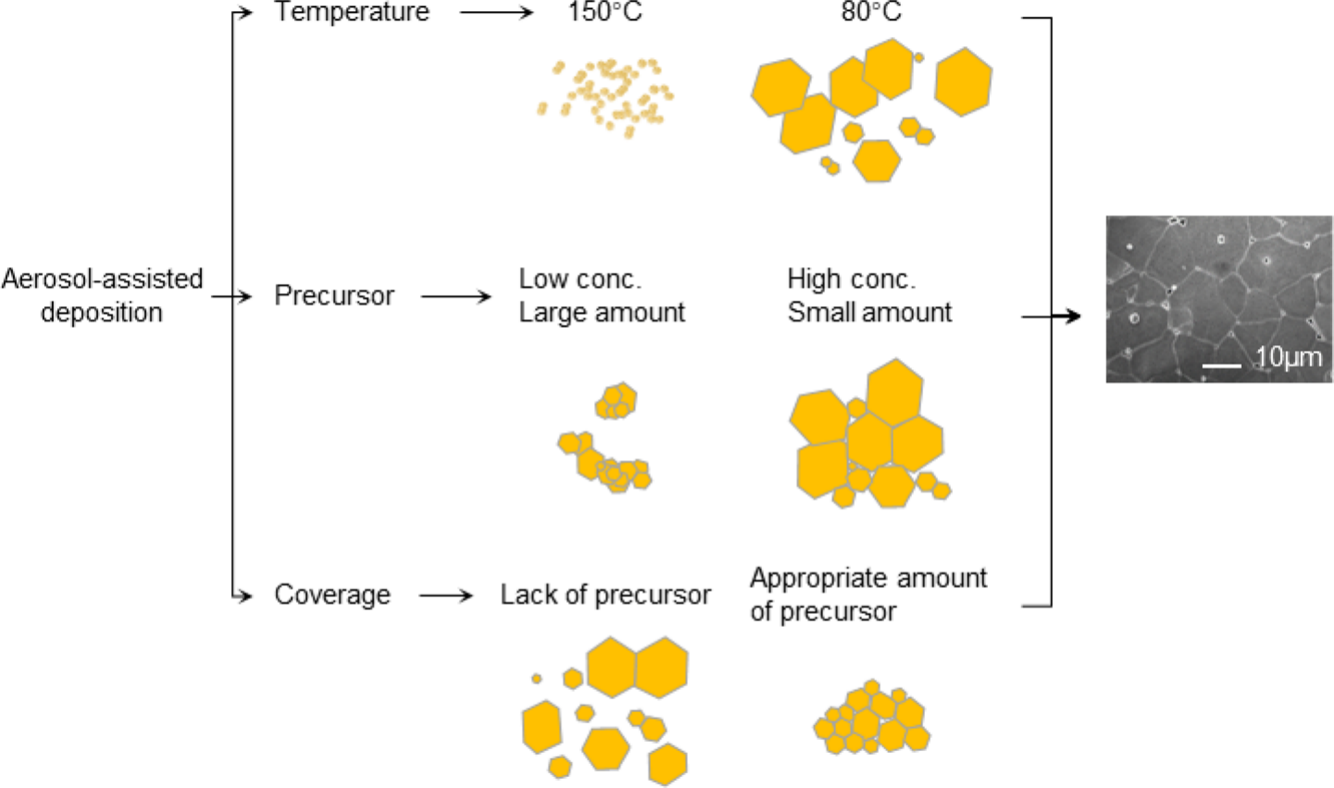
Scheme of the effect of different aerosol-assisted deposition parameters. Better morphology was achieved with the parameters on the right.

Powder samples of the perovskites were made by reaction of methylamine or cesium carbonate with TeO_2_ dissolved in aqueous hydroiodic acid. A precursor solution was obtained by dissolving the perovskite powder in a 4:1 v/v DMF:DMSO mixture, and growth of perovskite thin films was achieved by one-step aerosol-assisted deposition. The precursor solution was then nebulized inside a flask, and then the resultant aerosol was transported into the reaction chamber by N_2_ carrier gas (see Figure S1). Standard glass slides (2.5 × 2.5 cm^2^) and ITO-coated glass slides (2 × 2 cm^2^) were used as substrates. The deposited film quality was investigated as a function of temperature, precursor concentration, flow rate, aerosol concentration (nebulizer power), substrate, and precursor mass as summarized in Figures 1 and S2–S7 in the SI.

The deposition temperature was varied between 50 and 250 °C as tellurium perovskites usually decompose above 250 °C.^9^ High temperature (>100 °C) was found to provide a powdery film with poor coverage on the surface, while extensive coverage and better attachment were achieved at lower temperature. Optimal coverage was found at 80 °C for MA_2_TeI_6_ and 50 °C for Cs_2_TeI_6_, under which conditions the face-centered cubic structure perovskite crystals grow in a hexagon-shaped habit of typically 50 μm dimension (Figures 1 and S3).

The coverage of the deposited film was strongly correlated with the precursor volume and concentration, as demonstrated by the thin films shown in Figures 1 and S4 depicting representative images of film growths. The perovskite solution concentration was tested between 0.025 and 0.25 M, and the volume of the precursor was between 1 and 2 mL. We found that the concentration of the precursor is important: growth with a large amount of low concentration precursor resulted in a rough surface, while the same quantity of precursor in high concentration solution provided a better coverage of the substrates. Uniform growth of thin films can be achieved with near-saturated solutions, which were applied to verify the influence of other parameters.

Furthermore, we found that the film quality was a sensitive function of the flow rate, and it was tested between 200 and 800 mL/min. Qualitatively, a low flow rate produces inhomogeneous films with a gradient in coverage (Figure S5), and large flow rates tend to produce no deposition at all. An optimal flow rate should transport enough precursor to the reaction chamber and evenly cover the substrate, and it was found to be 300 mL/min for both MA_2_TeI_6_ and Cs_2_TeI_6_ in our setup. Similar to the effect of flow rate, the nebulizer power influences the nucleus deposition rate and growth speed with rough or patchy growth at extremes with an optimal value at intermediate levels.

**Figure 2:**
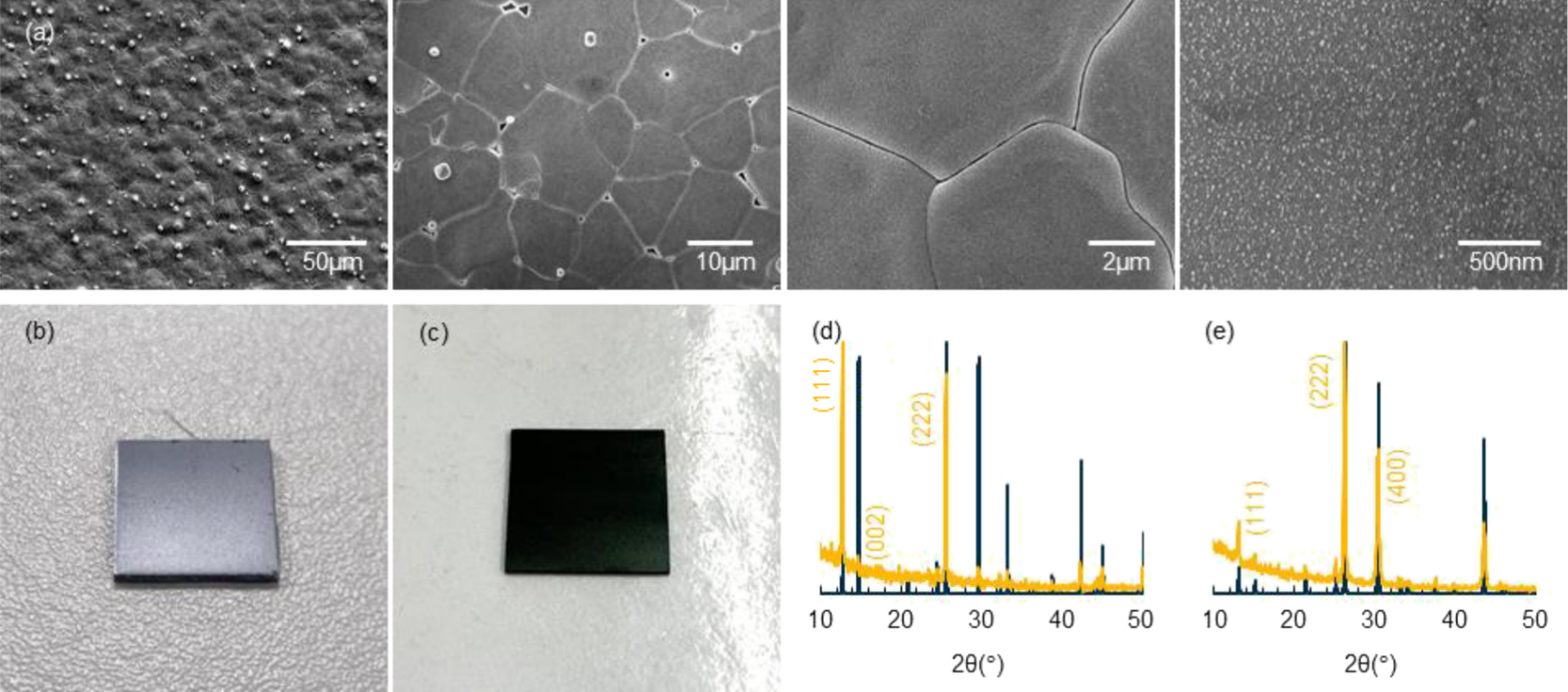
SEM images of (a) MA_2_TeI_6_ perovskite thin films deposited with optimal AACVD parameters; image of (b) MA_2_TeI_6_ cut into ∼1.25 × 1.25 cm^2^ and (c) Cs_2_TeI_6_ thin film in ∼2.5 × 2.5 cm^2^. Normalized XRD patterns of (d) MA_2_TeI_6_ and (e) Cs_2_TeI_6_ thin film grown on microscope glass slides by aerosol-assisted deposition (yellow) compared with crystallographic information files (cif) (blue).^5^

At optimal conditions, the perovskite seeds nucleated uniformly covering the substrate, and the flow provided a reasonable growth speed to maximize the coverage. Substrates were found to have little influence on the perovskite morphology using this deposition method (Figure S6). Preferentially oriented, continuous perovskite thin films were produced as shown in Figure 2. GIXRD showed only peaks corresponding to cubic MA_2_TeI_6_ or Cs_2_TeI_6_, with strong preferred orientation toward (111). At this low deposition temperature, our one-step aerosol-assisted deposition should be suitable for other halide perovskites and remove the concerns of high-temperature decomposition or limitation in the substrate size.

### Electrochemistry and Photoelectrochemistry Performance

**Figure 3:**
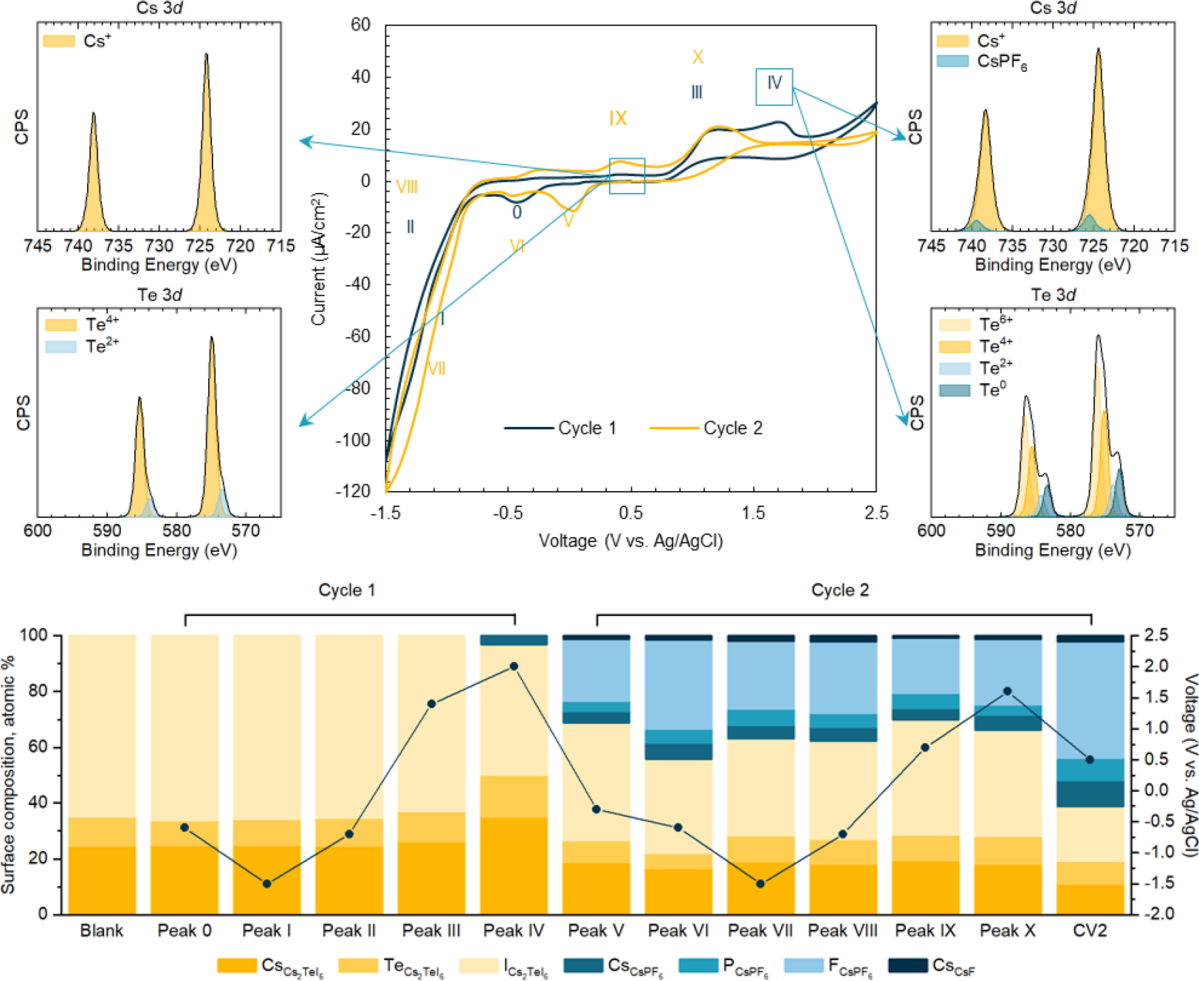
Top, cyclic voltammogram of the Cs_2_TeI_6_ film from 0.5 to −1.5 and 2.5 V (vs Ag/AgCl), recorded in DCM/TBAPF_6_ at a scan rate of 5 mV/s in the dark. XPS spectra of Cs_2_TeI_6_ films: (top left) as-synthesized; (top right) after peak IV in a partial CV scan. All the other XPS spectra are shown in the SI. Bottom, composition changes to the electrode surface measured by ex situ XPS at different points in the CV process.

Tellurium-containing perovskites exhibit strong visible light absorption and stability under ambient conditions (Figures S8 and S9).^5,34^ To study the electrochemical and photoelectrochemical performance, we used the thin film of Cs_2_TeI_6_ and MA_2_TeI_6_ grown on ITO substrates. Since the DCM electrolyte and blank ITO substrate offer a wide electrochemical window,^13,17^ all the redox peaks in CV measurements shown in Figure 3 are attributed to perovskite materials, which are stable in DCM at zero potential (Figures S10 and S11).

Fresh Cs_2_TeI_6_ samples were used in CV measurements to reduce the effect of moisture and surface oxidation as much as possible (Figure S12). While the CV was initially scanned from 0.5 to −1.5 V (vs Ag/AgCl), a small reduction peak appears first at −0.5 V vs Ag/AgCl (denoted as peak 0), and then the redox reactions (peak I, II) are assigned to electrolyte until two oxidation peaks (peak III, IV) appear while scanning anodically to 2.5 V vs Ag/AgCl. The reduction pairs of peaks were observed during the second cycle (denoted as peak V and VI respectively). In the second cycle, an extra oxidation peak is observed at 0.4 V (vs Ag/AgCl, peak IX), and a similar oxidation reaction (peak X) occurs at the same position as peak III.

To understand the redox processes, partial CV scans were undertaken, and then samples were immediately transferred for XPS to study the valence state change of each element. The XPS spectra of as-synthesized samples were calibrated based on the adventitious carbon peak set to a binding energy (BE) of 285.0 eV. In the as-presented sample, the Cs 3d peak appears symmetric, suggesting a single environment. The most intense feature of the Te 3d_5/2_ envelope at 574.9 eV is believed to be Te^4+^ (Figure 3). The small component at 573.6 eV is assigned to the Te^2+^ (Te^2+^:Te^4+^ = 0.16:1). Metallic tellurium is reported at 573.0 eV and Te^6+^ is typically found at a higher BE (576.0 eV) than the peaks we observe.^35^ This observed mix-valency is believed to be related to beam damage occurred in XPS measurements, which we discuss further in the supporting information (Figure S13).^36^

In the as-presented sample, the I 3d spectrum shows a main, symmetric feature for each spin orbit component. The largest I 3d_5/2_ component is at 618.7 eV for the as-synthesized sample and we assign this as iodide ions in Cs_2_TeI_6_, while the other higher BE peaks are likely loss features (which are observed in other elements in this study too, Figure S14) rather than additional iodine chemical states.^37^ Thus we interpret this spectrum as representing a single iodine environment corresponding to iodide ions in Cs_2_TeI_6_. The Cs 3d spectrum consists of symmetrical peaks at binding energies of 724.2 and 738.1 eV assigned to Cs^+^ in Cs_2_TeI_6_. The composition of the as-presented film is shown in Table S1 and is close to the expected Cs_2_TeI_6_ stoichiometry, but slightly iodine-deficient.

XPS shows that during the redox process, significant surface chemical changes occur. Due to the complex carbon environment after electrochemistry measurements, calibration to C 1s was not reliable (see Figure S14), so all spectra were calibrated based on the main I^–^ peak position set at 618.7 and 630.2 eV.^13^ All the spectra were deconvoluted and quantified as shown below and in Table S1 and Figure S15.

Chemical change is most apparent in the Te 3d signal. After the oxidation process, Te 3d_5/2_ spectra show additional oxidized peaks, interpreted as Te^6+^, at around 576.0 eV. The Te^6+^ peaks increase in intensity after oxidation reactions and decrease after reduction. On the contrary, lower binding energy Te 3d components are seen when negative potential is applied, corresponding with the increased peak area at lower BE (Figure S16 and Table S2). It is worth noting that TeI_4_ can be dissolved in the electrolyte as proved by its absorption spectra in Figure S18; thus tellurium may not be fully recovered after electrochemical cycling.

The iodine and cesium peaks show more minor differences in line shape during cycling, which implies that the observed redox processes are most associated with the tellurium in this compound (see the SI). At CV peaks III and IV, the iodine 3d spectrum broadens and can be fitted with a lower BE component. Up to CV peak V, i.e., during the first CV cycle, the Cs 3d signal is almost unchanged in both the position and width. In the second cycle (peak V and beyond), a higher BE component is clearly visible on the Cs 3d peaks.

Changes to composition during cycling are shown in Figure 3 and can be used to complete the picture of surface chemical changes. The initial composition of the electrode surface is close to the expected Cs_2_TeI_6_ stoichiometry. The composition remains fairly constant up to peak III, after which the Cs:I ratio begins to increase, indicating iodine loss from the surface. The Cs:I atomic ratio increases to 1:1.1 after peak IV and falls slightly after the subsequent negative voltage sweep to 1:1.7 by peak V. Subsequent cycling sees the iodine content fall still further. This suggests that iodine is dissolved in solution during this initial cycle, either due to I^–^ oxidation to soluble triiodide ion (peak IV) or dissolution of TeI_4_; the dissolution of iodine and tellurium is supported by the absorption spectra of electrolyte (Figures S17–S20). By the start of the second cycle, there is a significant amount of F and P present on the sample surface, which we attribute to the presence of CsPF_6_, formed from the reaction of the Cs_2_TeI_6_ with the TBAPF_6_ salt present in the electrolyte. SEM, EDX, and XRD analysis (Figures S21, S22, and S24) corroborate the presence of CsPF_6_ on the surface after cycling. We therefore assign the Cs 3d_5/2_ photoemission peaks at 725 eV to CsPF_6_ The quantity of CsPF_6_ varies slightly over cycle 2, with a maximum at Peak VI.

In summary, we find that at the end of the first CV cycle, a layer of CsPF_6_ forms at the surface of the electrode. This is accompanied by some loss of iodine and tellurium to the solvent, likely as I_3_^–^ and TeI_4_. The surface CsPF_6_ layer may act to passivate the film from further electrochemical corrosion (Figures S21–S24), as previously reported in CsPbBr_3_.^13^

**Figure 4:**
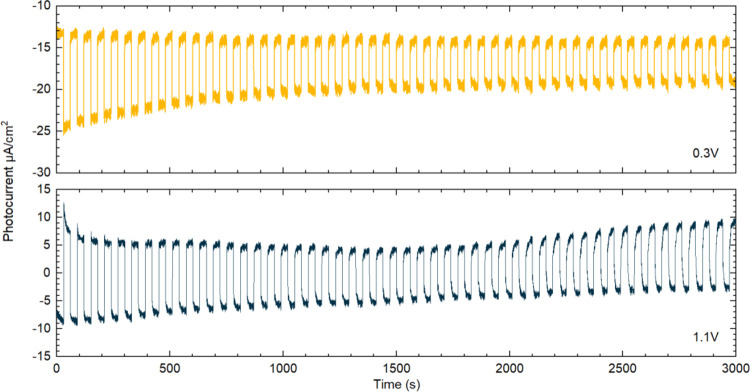
Chopped-light measurements of Cs_2_TeI_6_ at 0.3 and 1.1 V (vs Ag/AgCl) in DCM/TBAPF_6_. All measurements started in the dark.

The photochemical performance of Cs_2_TeI_6_ was investigated with chopped-light measurements carried out at 0.3 and 1.1 V (vs Ag/AgCl) as shown in Figure 4. Both measurements were started in the dark and the shutter opened after 30 s. An increase in photocurrent is observed at 1.1 V (vs Ag/AgCl) while the current drops at 0.3 V when the shutter is open. Upon illumination, the electrons are excited to the conduction band and injected into ITO, while holes are transferred to electrolyte. The photocurrent density of Cs_2_TeI_6_ in DCM at the beginning is 16 μA/cm^2^ at 1.1 V and 11 μA/cm^2^ at 0.3 V (vs Ag/AgCl), which is promising compared with Cs_2_PtI_6_ (0.2 μA/cm^2^) in the same electrolyte.^17^ However, degradation of the surface leads to a slight decrease in excited photocurrent with the increasing time. The photocurrent becomes stable or even slightly increases at later time, which may be related to the decompositions dissolved in the electrolyte.

Similar experimental procedures were carried out on MA_2_TeI_6_ (all data are provided in Table S3 and Figures S28–S32). The CV measurement is similar to Cs_2_TeI_6_, suggesting similar redox reactions. Previously, we assumed that most of the Cs^+^ was not dissolved in the DCM electrolyte due to the formation of CsPF_6_ on the surface, which protects the perovskite film beneath from further electrochemical corrosion. However, the methylammonium cation can be easily dissolved in DCM; thus it is unable to prevent the corrosion. XRD shows a more obvious existence of tellurium metal after reduction in MA_2_TeI_6_ (Figures S24 and S30), while SEM and EDX cannot find similar morphology and F/P-rich distribution compared to Cs_2_TeI_6_, which supports the protective effect of CsPF_6_. In addition, MA_2_TeI_6_ thin films exhibit lower stability to the air or Ar ion during XPS measurements, evidenced by more chemical environments observed in the blank film. However, the surface decomposition of MA_2_TeI_6_ does not affect the bulk material stability as no extra peaks are observed in the XRD pattern.

To better understand the redox processes on the surface, it is important to determine the flat band potential (*V*_FB_). The *V*_FB_ was first measured via Mott–Schottky (MS) analysis.^38^ The measurement of capacitances comprises both the space charge region and the charge transfer at high frequencies, while the ion movement and electrolyte diffusion dominate at low frequencies.^17^ Thus, the frequencies are selected between 10 and 0.1 Hz to avoid stray capacitances, which are lower than usual as DCM was used as the electrolyte.^17,38^ The MS plot was constructed from EIS data collected at various frequencies as shown in Figure S25. The intercept of the linear portion is determined as *V*_FB_ according to the MS equation:^39^
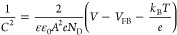
where *V* is the applied potential, *e* is the electronic charge, *N*_D_ is the number of donors, *k*_B_ is the Boltzmann’s constant and *T* is the absolute temperature; *C* and *A* are the interfacial capacitance and area, respectively. Therefore, the flat band potential is found to be around 0.38 V vs Ag/AgCl. However, MS measurement is based on multiple assumptions, and hence, the *V*_FB_ result has a large uncertainty in practice.^38,40^

The open circuit potential *V*_OCP_ was measured as 0.412 V in the dark increasing to 0.469 V (vs Ag/AgCl) under illumination (Figure S26), conforming to a p-type semiconductor material.^9,38^ Theoretically, the *V*_OCP_ will reach the flat band potential under sufficient illumination. However, it is difficult to achieve equilibrium with photogenerated carriers, and this measurement will be affected by defects or the oxygen dissolved in the electrolyte.^38,40^ Therefore, *V*_FB_ is deduced to be greater than 0.469 V (vs Ag/AgCl).

The flat band energy can also be identified as the point where the inversion of anodic and cathodic photocurrent occurs.^38^ A chopped-light LSV measurement was carried out on Cs_2_TeI_6_ film from 2.5 to −1.5 V (vs Ag/AgCl) with a slow scan rate (2 mV/s), as shown in Figure S27. The photoelectrochemical photocurrent switching effect controlled by external potential is observed. Cs_2_TeI_6_ generates anodic photocurrent in potential range from 2.5 to 0.6 V vs Ag/AgCl while cathodic photocurrent at lower potentials. This transient response indicates the band bending of the material. Therefore, *V*_FB_ is identified between 0.55 and 0.63 V vs Ag/AgCl where the direction of band bending changes. Taking all the results into consideration, subsequent discussion is based on *V*_FB_ = 0.55 V vs Ag/AgCl. Based on the above observations, the main redox reaction is likely to be:

Oxidation
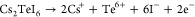

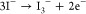


Reduction:
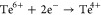

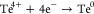

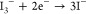


**Figure 5:**
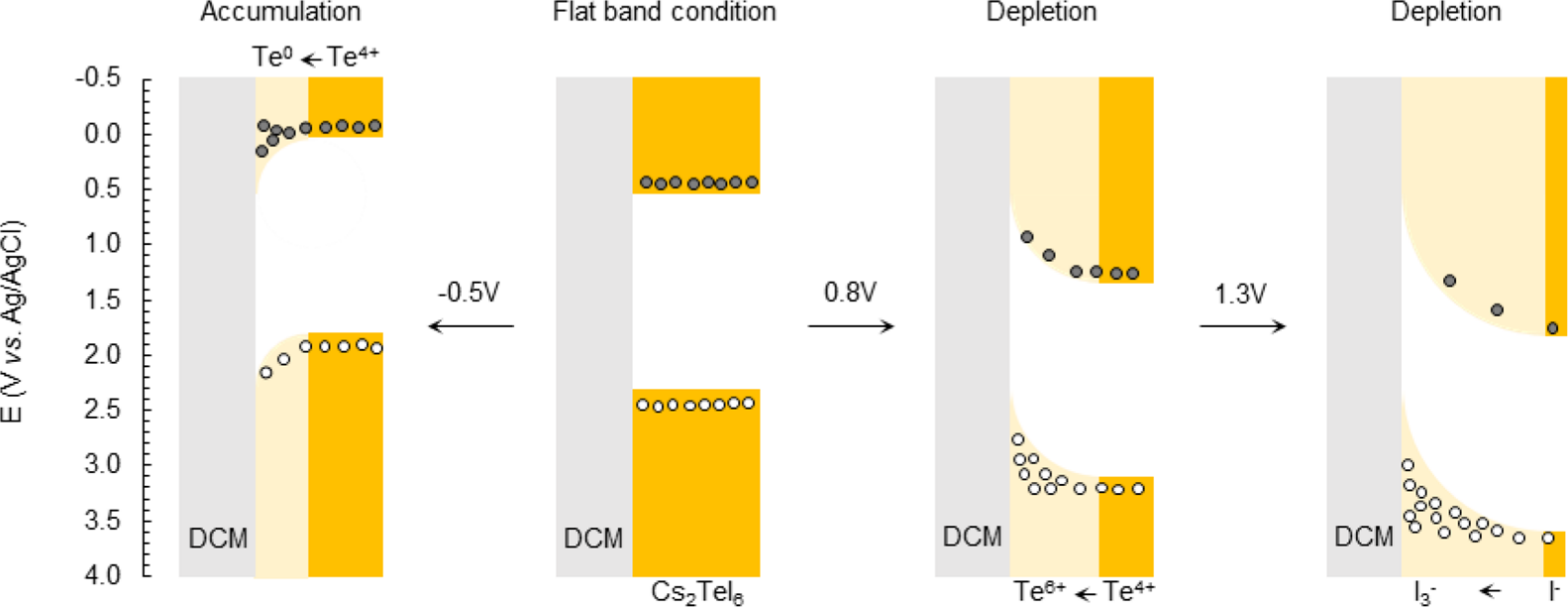
Proposed band bending of Cs_2_TeI_6_ at various applied potentials in DCM. Yellow represents the conduction band and valence band of Cs_2_TeI_6_, while light and dark shades correspond to the space charge layer and bulk material, respectively. Gray represents the DCM electrolyte; dark gray and white circles represent electron and hole carriers, respectively.

To discuss this further, we present a proposed band alignment diagram of Cs_2_TeI_6_ depicted in Figure 5. Depletion and accumulation regions are formed upon the application of positive and negative potentials (vs Ag/AgCl). At an applied potential of 1.3 V, the band bends down which enriches the holes in the valence band at interface and depletes the electron in the conduction band. On the other hand, at −0.5 V (vs Ag/AgCl) where the accumulation region forms at the interface, the holes are depleted at the valence band while the electrons will accumulate at the conduction band. It is worth noting that as iodine has weak electronegativity, the Te–I chemical bonding will not be fully ionic; thus the oxidation state of tellurium in the perovskite structure should be lower than 4+. This can explain the extra oxidation peak in XPS at higher BE.

## Conclusions

In summary, we have applied low-temperature, one-step aerosol-assisted deposition to grow A_2_TeI_6_ perovskite thin films. Preferred-oriented growth (111) of face-centered cubic structure perovskite was confirmed by SEM and GIXRD. This low-temperature processing allows large-scale deposition of perovskite thin films and a flexible choice of substrate.

The electrochemical and photoelectrochemical performances of Cs_2_TeI_6_ and MA_2_TeI_6_ have been studied, and the mechanism of the redox reaction in the DCM electrolyte was analyzed. Cs_2_TeI_6_ shows a larger photocurrent density than Cs_2_PtI_6_, which provides more possibilities for it to work as a photocatalyst. Moreover, the photoelectrochemical photocurrent switching effect was observed in chopped-light LSV measurement, indicating that Cs_2_TeI_6_ can be potentially applied both as the anode and cathode in the photocatalytic reaction. An SEI consisting of CsPF_6_ was found on the surface of the electrode after contact with DCM and TBAPF_6_. In contrast, an MA_2_TeI_6_ electrode is incapable of forming a protective layer: MAPF_6_ is not formed on the surface and the film rapidly degrades. This layer may be the reason that Cs_2_TeI_6_ is more stable than the MA_2_TeI_6_ analogue.

## Supporting Information Available

Further characterization data, full XPS data, and quantification (PDF)
