# Supplementary material for: Solid Electrolyte Interphase Formation in Tellurium Iodide Perovskites during Electrochemistry and Photoelectrochemistry
Source: ACS Appl Mater Interfaces. 2023 Jul 24;15(30):37069–76. doi: 10.1021/acsami.3c07425 (PMC10401509; doi:10.1021/acsami.3c07425)
Supplement: Supplementary file 1 — am3c07425_si_001.pdf [file am3c07425_si_001.pdf]

Supporting Information

Solid Electrolyte Interphase Formation in Tellurium Iodide Perovskites during Electrochemistry and Photoelectrochemistry

Yuhan Liu,[1] Yuting Yao,[1] Xinyue Zhang,[1] Christopher Blackman,[1] Robin S. Perry[2] and Robert G. Palgrave[1,*]

[1] Department of Chemistry, University College London, Christopher Ingold Building, 20 Gordon Street, London, WC1H 0AJ UK

[2] London Centre for Nanotechnology and Department of Physics and Astronomy, University College London, 17-19 Gordon Street, London, WC1H 0AH UK

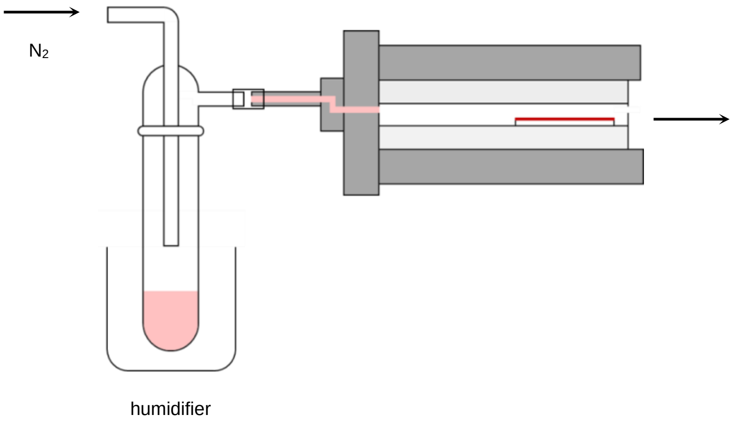

Figure S1 Scheme of applied aerosol-assisted deposition process.

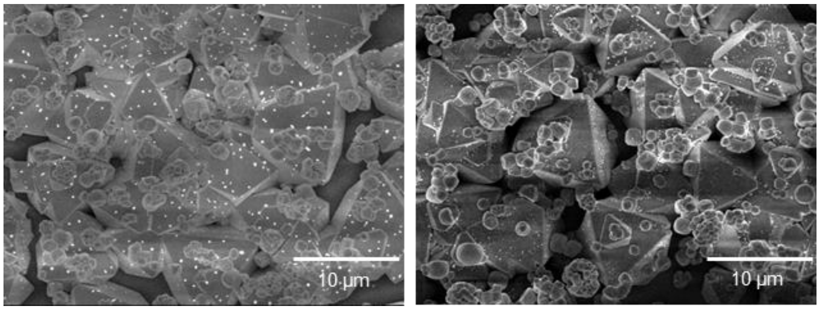

Figure S1 SEM images of perovskite thin films with (left) 10s and (right) 20s gold coating. Number of bright spots increased with longer coating time so they are aggregated gold nanoparticles that cannot spread on the surface of perovskites due to unknown reason.

SEM images in Figure S2 demonstrate the different perovskite growth trends observed at various temperatures. Deposition at temperatures above 100 °C was found to provide a powdery film with poor coverage on the surface, as shown in Figure S2. The perovskite particles exhibit hexahedron-like shapes that are uniform in size, typically ~1 μm at 150 °C, but without any preferred orientation. The reason may be related to the high solvent evaporation rate at elevated temperatures causing rapid deposition of small particles that subsequently poorly adhere to the substrate. In contrast, more extensive coverage and better attachment were achieved at lower temperature deposition, as shown in Figure S2. In practice, the inorganic perovskite film is found to be less preference-oriented than hybrid perovskites. This could be solved by lowering the deposition temperature. However, lower temperature will also deteriorate the precipitation from solvent, in which case a solvent with lower boiling point should help.

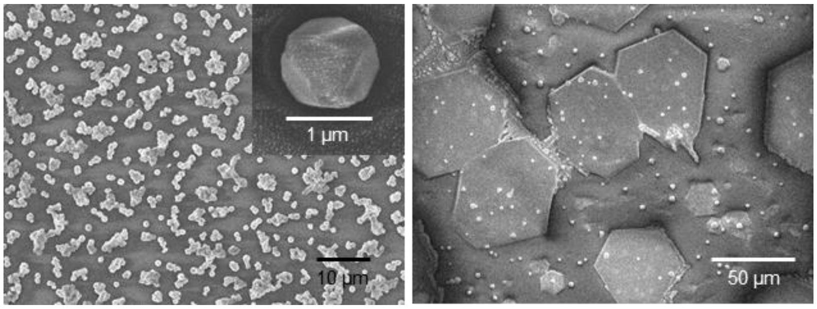

Figure S2 SEM images of MA_2TeBr_6 thin films deposited on microscope glass slides at (left) 150°C and (right) 80°C. Lower temperature can encourage the aerosol deposit as thin film while high temperature will generate perovskite particles directly.

The perovskite solution concentration was tested between 0.025 M to 0.25 M, and the volume of precursor was between 1 and 2 ml. A low concentration, low volume precursor (Figure S3(a)) produces films barely covering the surface due to mass limitations, while a larger volume of precursor (Figure S3(b)) has improved coverage for the same concentration. The effect of concentration can be viewed in Figure S3(b,c); for an identical volume of precursor, improved

coverage with a different morphology is achieved using 0.05 M solution. Moreover, a high concentration, low volume of precursor (Figure S3(d)) promotes large perovskite ‘flakes’ with a well-presented hexagonal habit on the substrate.

Although simply increasing the amount of precursor solution can provide more material and hence coverage, it did not fully cover the substrate in practice. Repeated growths attempts using low concentration precursors had an interesting effect: the perovskite flakes tend to stack upon each other, resulting in a rough surface. In other words, no new nucleation sites were generated, and existing crystals preferentially nucleated stacked growth. In contrast, a higher concentration precursor may provide enough nuclei on the substrate in the initial stages of deposition, which maximize the coverage to grow a perovskite thin film. From our exhaustive tests, we conclude that a small amount of high concentration precursor can provide the best coverage of the substrates. We found the optimal concentration to be 0.2 M for MA_2TeI_6 and MA_2TeI_6 (near saturated solution) and these conditions were applied to verify the influence of other parameters.

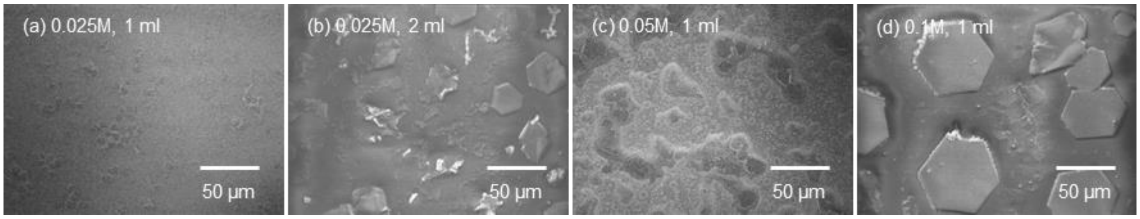

Figure S3 SEM images of MA_2TeI_6 thin films on microscope glass slides using different precursor concentration.

The flow rate in the aerosol-assisted deposition process is known to affect the morphology of perovskite films, and it was tested between 200 and 800 ml/min. As the viscosity of DMF/DMSO is higher than methanol or other common AACVD solvents, the volume of nebulized solvent is comparatively low. Thus, the amount of aerosol can be assumed to be constant for each growth. We note that a higher flow rate will transport more aerosol/N_2 per unit of time; thus, the proportion

of aerosol should be reduced accordingly. Figure S4 shows the different morphology of perovskite thin film due to an unsuitable flow rate. Although the right part exhibits a dense coverage of perovskite film, large hexagonal perovskite flakes are observed on the left because of precursor shortage. An optimal flow rate should transport enough precursor to the reaction chamber and evenly cover the substrate, and it was found to be 300 ml/min for both MA_2TeI_6 and Cs_2TeI_6.

With an optimized flow rate and a constant nebulizer power, the aerosol concentration can still vary due to the remaining volume of precursor solution inside the flask. As the height of the liquid level drops during growth, the volume of nebulized aerosol was observed to change over time. This affected the ratio of perovskite aerosol to carrier gas, feeding into changes in the thin film morphology. We applied a simple solution to this problem: to increase the nebulizer power over time to provide a stable aerosol concentration during the lifetime of the growth.

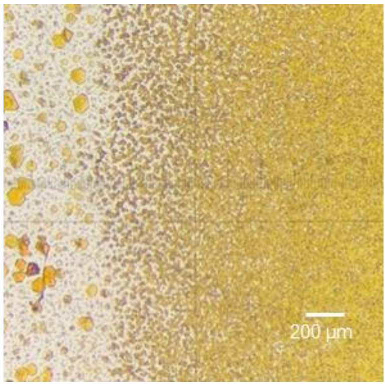

Figure S4 Images of MA_2TeBr_6 film deposition under optical microscope. The right part exhibits a dense coverage of perovskite film while large hexagonal perovskite flakes grow on the left due to lack of precursor.

Finally, to observe the effect of different substrates, both microscope glass slides and ITO-coated glass were tested. SEM images (Figure S5) indicate that the change of substrates has little effect on the perovskite morphology. Under the same deposition conditions, the perovskite follows the

same preference-oriented growth on top of the surface. However, a larger amount of precursor was required for the ITO-coated glass in practice to obtain a similar coverage as microscope glass slides.

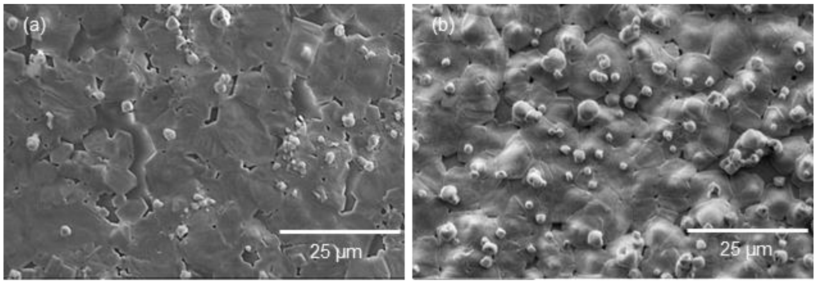

Figure S5 SEM images of MA_2TeI_6 deposited on (a) microscope glass slide and (b) ITO-coated glass slide.

The thickness of perovskite films can vary depends on the amount of precursor. The thickness of perovskite film increases with more precursor applied with the same concentration.

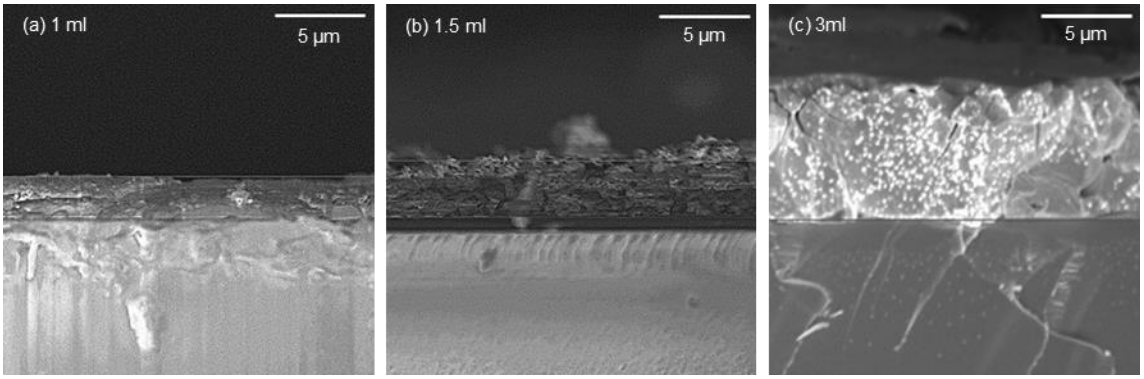

Figure S6 Cross-sectional SEM images of perovskite thin films deposited by AACVD. Film thickness can be adjusted by using different amount of perovskite precursor.

The perovskite absorption coefficients were calculated from UV-vis absorption spectra and film thickness as shown in Figure S7, which are similar to $MAPbI_3$.[1]

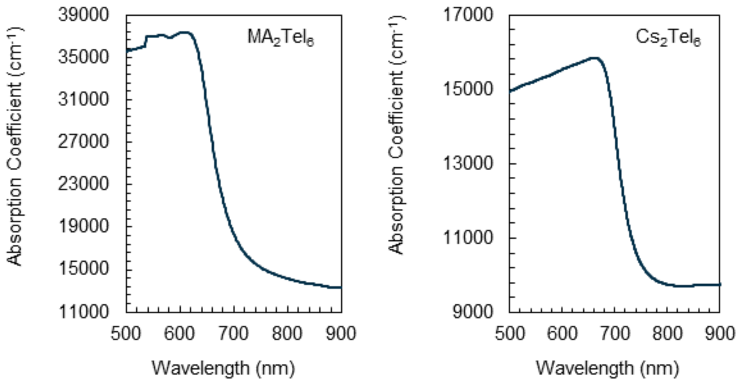

Figure S7 Absorption coefficient from UV-vis absorption data for MA_2TeI_6 and Cs_2TeI_6.

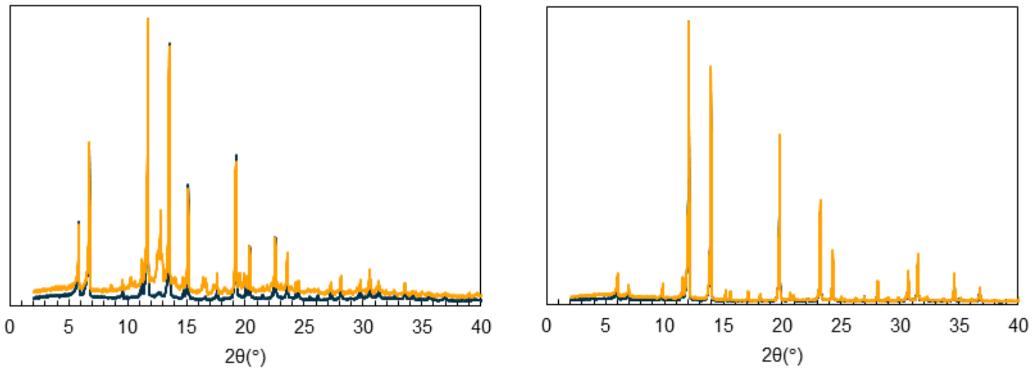

Figure S8 XRD patterns of (left) MA_2TeI_6 and (right) Cs_2TeI_6 before (blue) and after (yellow) dispersal in water for 1 minute.

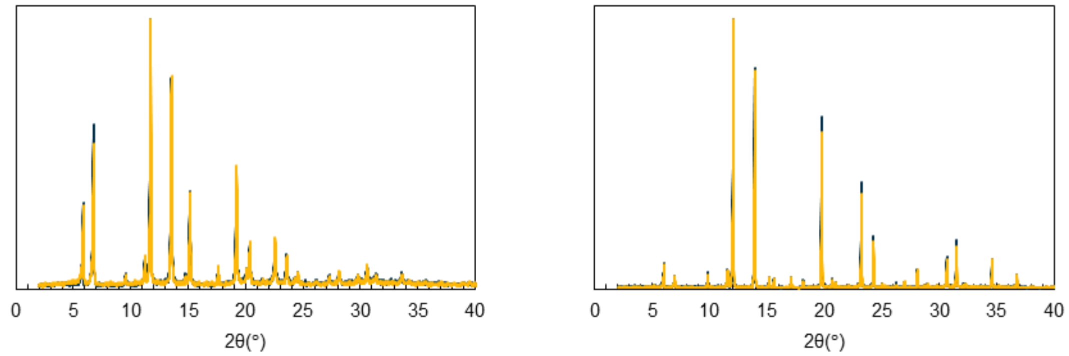

Figure S9 XRD patterns of (a) MA_2TeI_6 and (b) Cs_2TeI_6 before (blue) and after (yellow) dispersal in DCM for 2 hours. The materials remain stable.

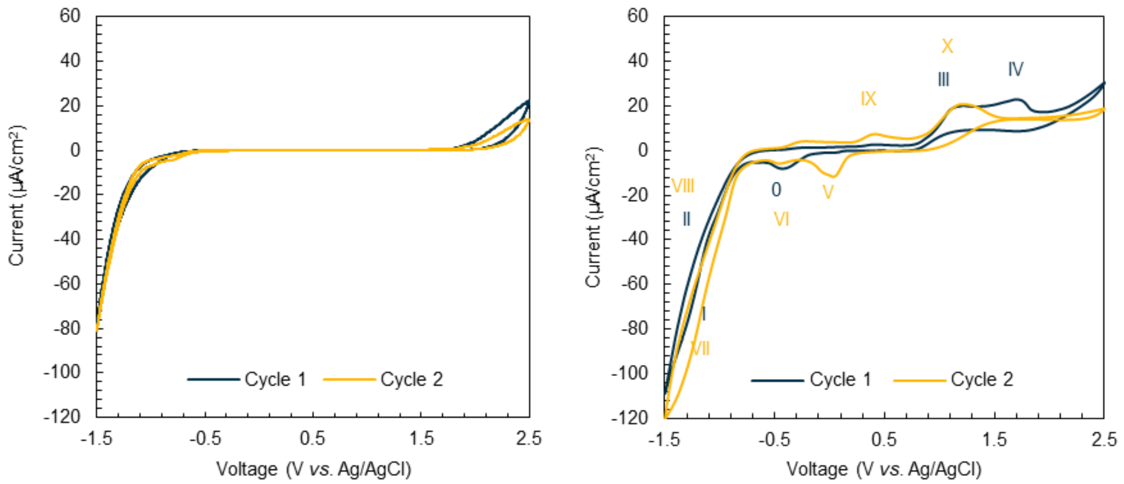

Figure S10 Cyclic voltammogram of blank ITO (left) and Cs_2TeI_6 film (right) *vs*. Ag/AgCl, carried out in DCM/$TBAPF_6$ in dark at a scan rate of 5 mV/s.

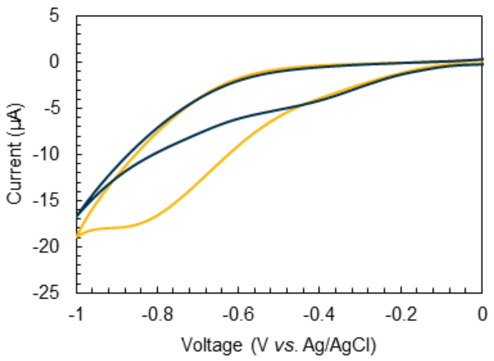

Figure S11 Cyclic voltammogram of Cs_2TeI_6 film exposed to the air for 0.5 hour (blue) and 5 hours (yellow), carried out in DCM/$TBAPF_6$ in dark at a scan rate of 5 mV/s. Therefore all the measurements were carried out in fresh.

X-ray beam damage is observed in XPS. Survey and core level spectra were collected 3 times at the same position on Cs_2TeI_6 film. Cs and I spectra remain the same peak shape across the scans, while increasing shoulders are observed at lower binding energy in Te core spectra. This may be because Cs_2TeI_6 is sensitive to X-ray that the electrons can reduce Te^{4+} to tellurium metal.[2,3] Therefore, in the following XPS analysis, an area scan was set up for tellurium and only 1 scan was taken on each data point to avoid the effect of beam damage as much as possible.

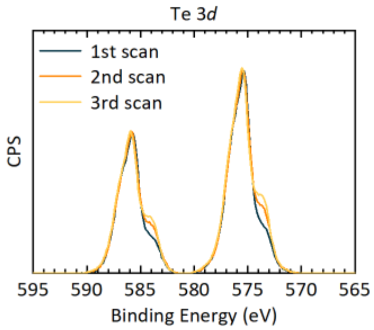

Figure S12 Overlapped, background subtracted XPS spectra of tellurium that observes beam damage.

XPS core level spectra are shown as measured in Figure S13. We observed extra peaks at higher BE in all the elements of perovskite, which are assigned as loss features and will not be further discussed. The organic electrolyte DCM and $TBAPF_6$ used in experiments generate a complex chemical environment of carbon after reactions as shown below, thus it is not feasible to calibrate the XPS data against adventitious carbon. However, although the peak area varies a lot across all the samples, the peak shape of iodine spectra does not have an obvious change. This may be because the environment of iodine on electrode remains the same. The highest peaks at 618.7 eV and 630.2 eV should be the I^- which exists in perovskite structures. The minor difference in iodine spectra indicates redox reactions can be hardly assigned to iodine oxidation state changes in the

perovskite structure. Thus, all the other samples were calibrated based on their I^- peak position due to the complex carbon environment after electrochemistry measurements.

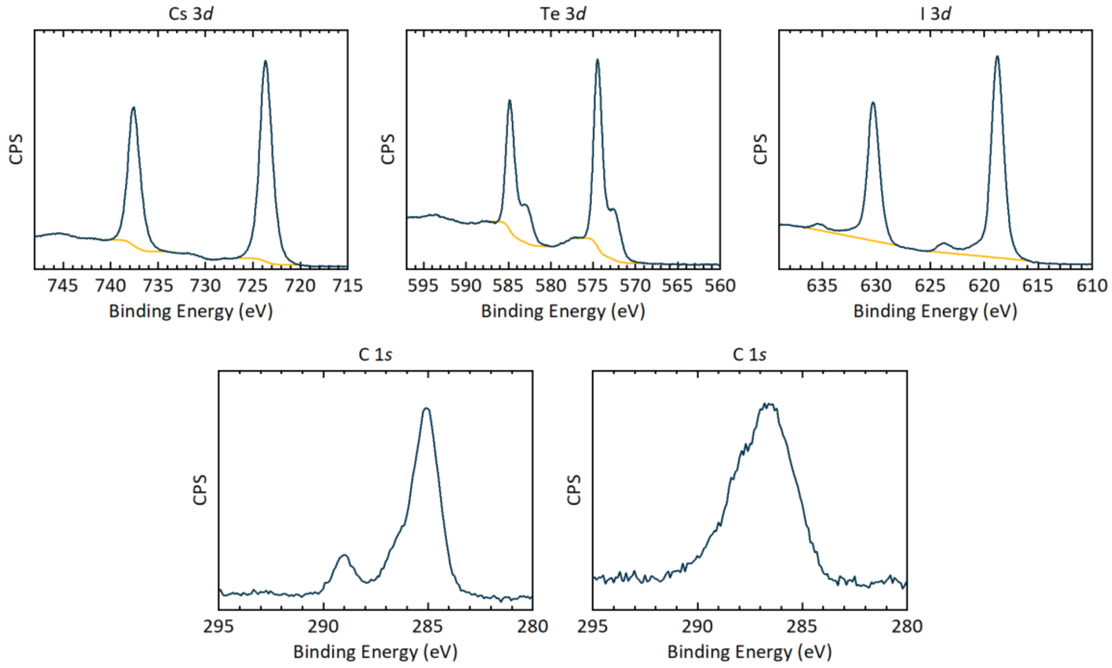

Figure S13 Top: XPS core level spectra of Cs (left), Te (middle) and iodine (right) as measured. Loss features are observed at higher BE in all elements, which are not included in further discussion. Background fitting is illustrated by yellow lines, the loss features of Cs and Te are excluded while I's is included for better fitting. Bottom: XPS spectra of C 1*s* before (left) and after (right) the electrochemical reactions.

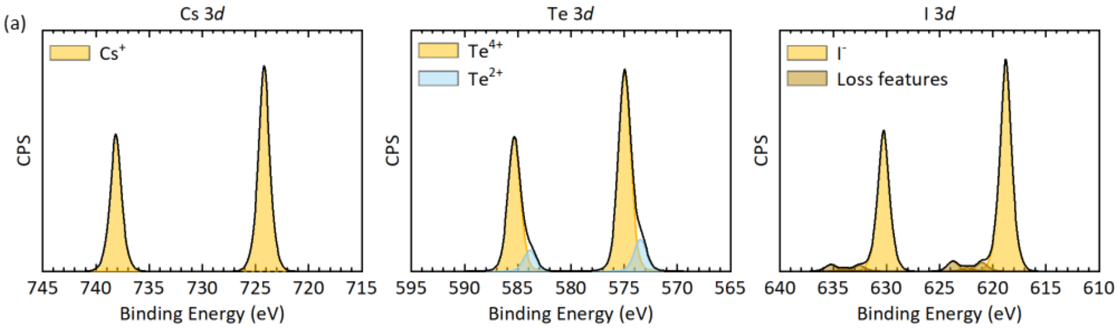

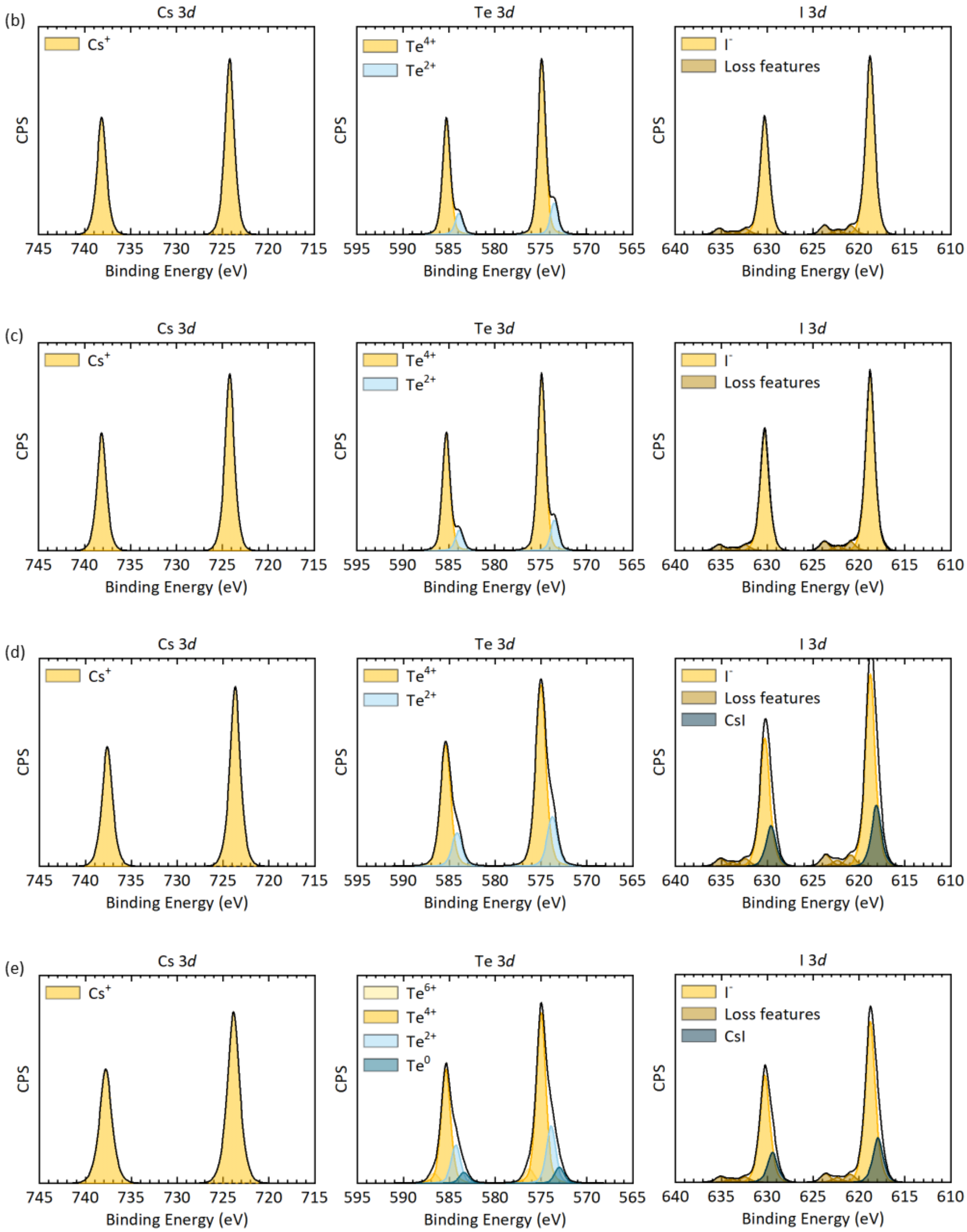
(b)
Cs 3d
Te 3d
I 3d
Cs+
Te4+
Te2+
I-
Loss features
CPS
Binding Energy (eV)
(c)
(d)
CsI
(e)
Te6+
Te0

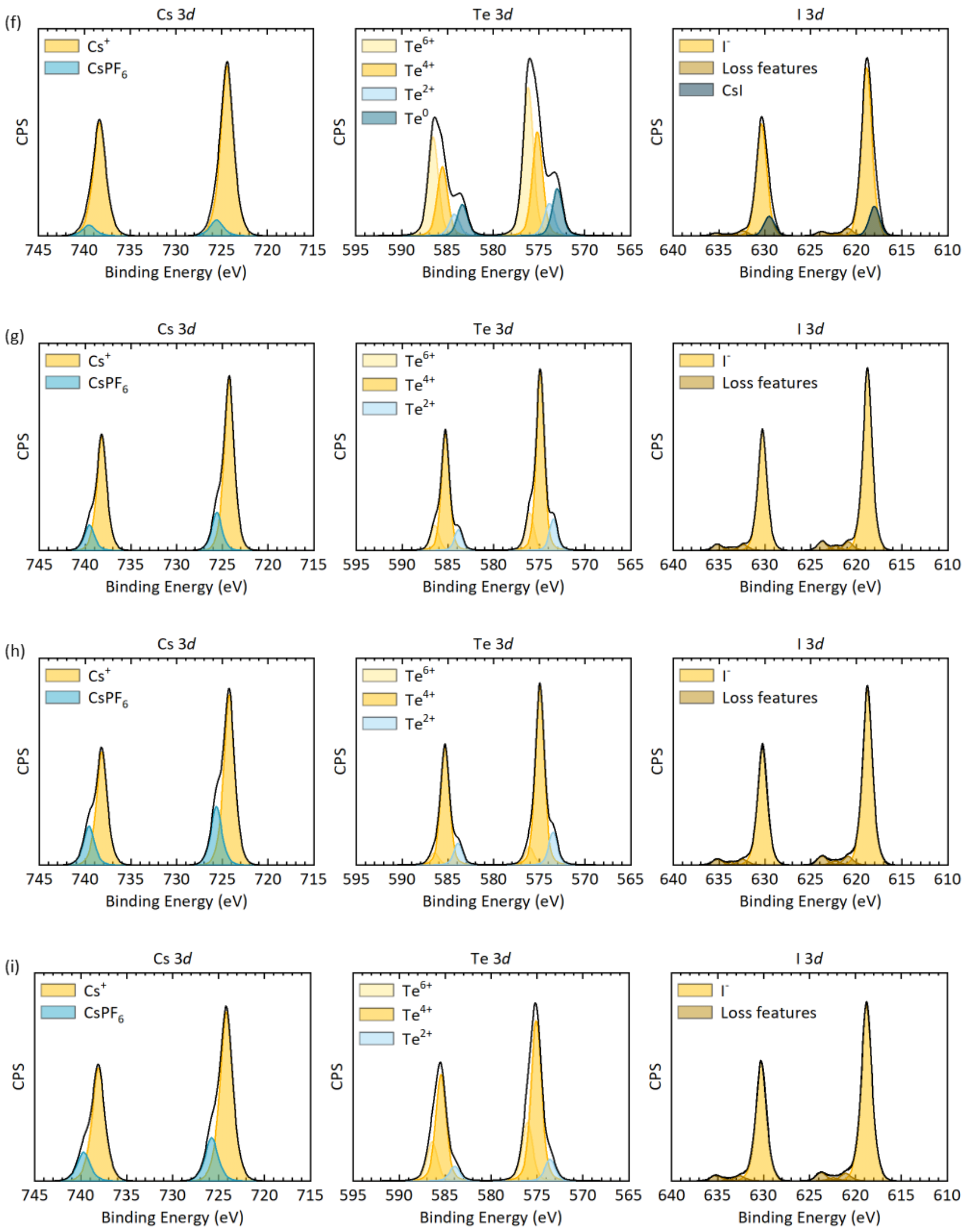
(f)
Cs 3d
Cs+
CsPF6
CPS
745 740 735 730 725 720 715
Binding Energy (eV)
Te 3d
Te6+
Te4+
Te2+
Te0
CPS
595 590 585 580 575 570 565
Binding Energy (eV)
I 3d
I-
Loss features
CsI
CPS
640 635 630 625 620 615 610
Binding Energy (eV)
(g)
Cs 3d
Cs+
CsPF6
CPS
745 740 735 730 725 720 715
Binding Energy (eV)
Te 3d
Te6+
Te4+
Te2+
CPS
595 590 585 580 575 570 565
Binding Energy (eV)
I 3d
I-
Loss features
CPS
640 635 630 625 620 615 610
Binding Energy (eV)
(h)
Cs 3d
Cs+
CsPF6
CPS
745 740 735 730 725 720 715
Binding Energy (eV)
Te 3d
Te6+
Te4+
Te2+
CPS
595 590 585 580 575 570 565
Binding Energy (eV)
I 3d
I-
Loss features
CPS
640 635 630 625 620 615 610
Binding Energy (eV)
(i)
Cs 3d
Cs+
CsPF6
CPS
745 740 735 730 725 720 715
Binding Energy (eV)
Te 3d
Te6+
Te4+
Te2+
CPS
595 590 585 580 575 570 565
Binding Energy (eV)
I 3d
I-
Loss features
CPS
640 635 630 625 620 615 610
Binding Energy (eV)

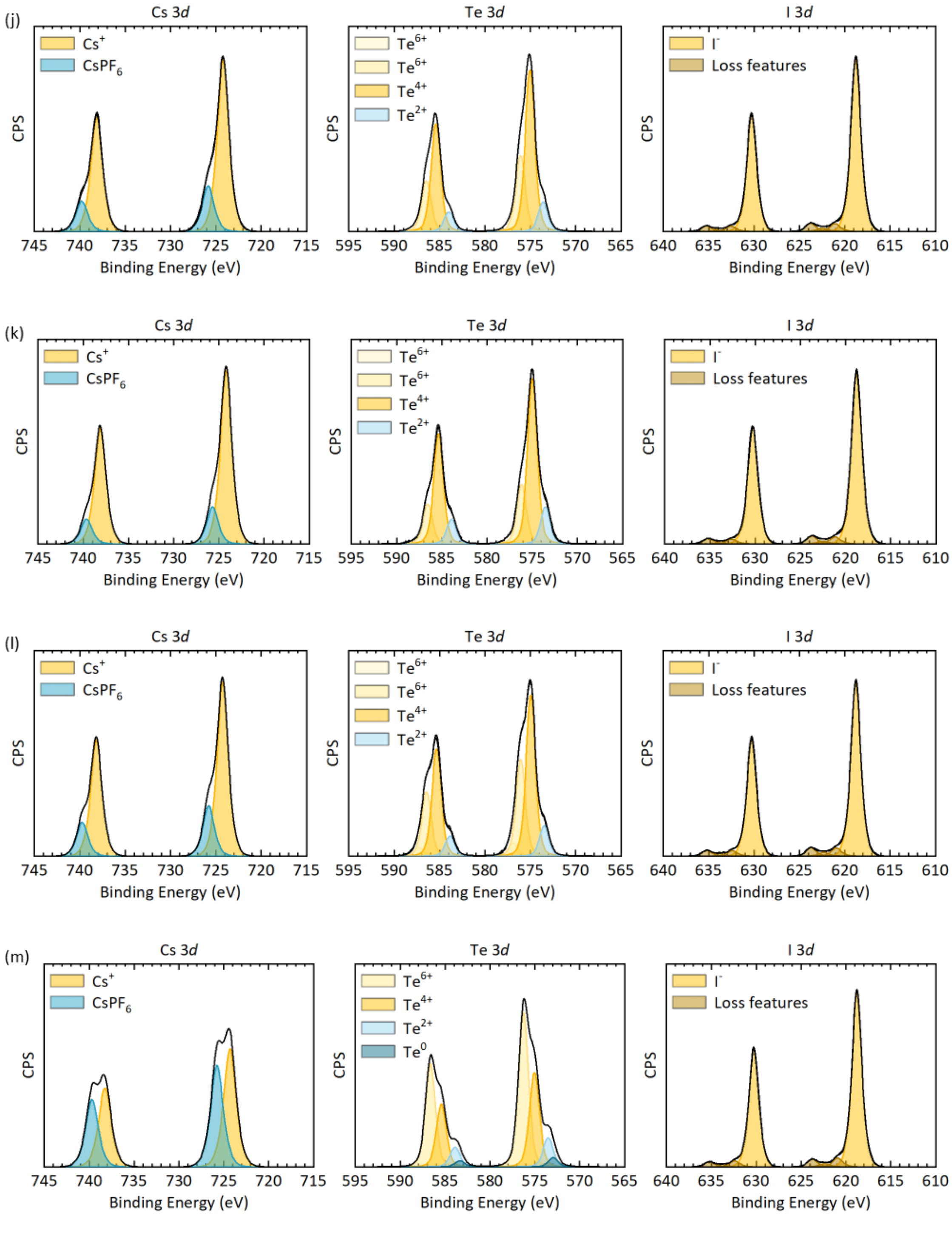

Figure S14 Background subtracted XPS core level spectra of Cs_2TeI_6 films: (a) as-synthesized; after (b-l) peak 0 to peak X in a partial CV scan and (m) after completing two CV cycles.

Table S1 Elemental ratio of Cs_2TeI_6 film surface before and after electrochemistry reactions

	Cs_2TeI_6			$CsPF_6$			$TBAPF_6$		CsF
	**Cs**	**Te**	**I**	**Cs**	**P**	**F**	**P**	**F**	**F**
Blank	24.6%	10.6%	64.9%	-	-	-	-	-	-
Peak 0	20.7%	7.5%	55.1%	-	-	-	2.1%	14.6%	-
Peak I	16.9%	6.4%	44.7%	-	-	-	5.0%	27.0%	-
Peak II	21.4%	8.5%	56.5%	-	-	-	1.7%	12.0%	-
Peak III	21.0%	8.7%	50.7%	-	-	-	2.0%	17.6%	-
Peak IV	24.3%	10.4%	32.2%	2.3%	-	-	5.4%	25.4%	-
Peak V	16.1%	6.6%	35.9%	3.6%	3.2%	18.8%	2.5%	12.4%	1.1%
Peak VI	15.5%	5.1%	31.6%	5.3%	4.7%	29.9%	2.1%	4.5%	1.3%
Peak VII	17.1%	8.3%	31.2%	4.4%	5.3%	21.7%	1.4%	8.9%	1.7%
Peak VIII	17.3%	8.3%	33.4%	4.6%	4.8%	24.2%	1.5%	3.9%	2.0%
Peak IX	15.7%	7.4%	33.5%	3.3%	4.3%	16.1%	3.0%	16.0%	0.6%
Peak X	15.0%	8.1%	31.5%	4.3%	3.1%	19.3%	3.5%	14.1%	1.0%
After 2 CV cycles	9.4%	7.0%	16.4%	8.1%	6.8%	35.3%	3.3%	11.8%	1.8%

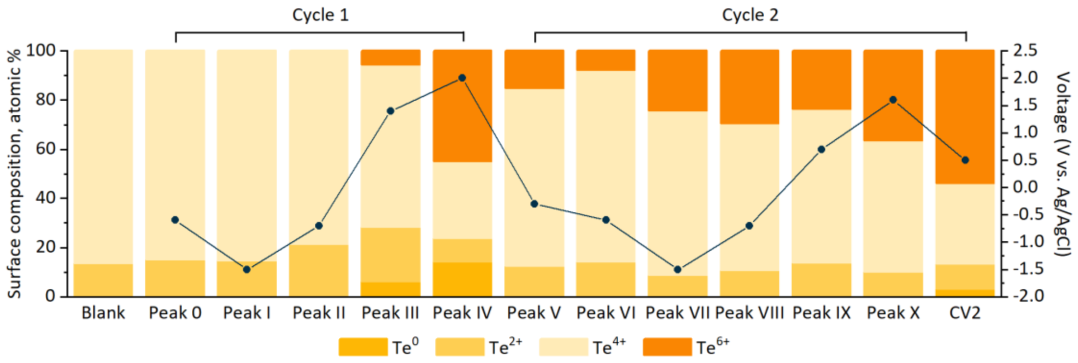

Figure S15 The change of tellurium chemical states at different points in the CV process.

Table S2 The relative ratio between different tellurium chemical states at different points in the CV process.

	Te^0	Te^{2+}	Te^{4+}	Te^{6+}
Blank	-	12.8%	87.2%	-
Peak 0	-	16.3%	83.7%	-
Peak I	-	15.4%	84.6%	-
Peak II	5.2%	21.2%	73.5%	-
Peak III	6.2%	22.2%	66.1%	5.6%
Peak IV	14.5%	9.6%	31.3%	44.5%
Peak V	-	13.2%	73.2%	13.7%
Peak VI	-	15.0%	77.9%	7.1%
Peak VII	-	9.4%	66.9%	23.7%
Peak VIII	-	11.4%	61.3%	27.3%
Peak IX	-	13.9%	63.7%	22.4%
Peak X	-	10.4%	56.6%	33.0%
After 2 CV cycles	1.9%	12.1%	32.7%	53.3%

The I 3d spectra of Cs_2TeI_6 after Peak IV is normalized against the intensity of blank sample as shown below. When the spectrum is normalized against the highest peak, which is allocated as the I^- in perovskite structure, the intensity of loss feature peak at 623.7 eV (blue) is about half of the blank sample (black). However, all the other samples' loss features have similar intensities. Thus, it is reasonable to assume that for the iodine in perovskite structure, the amount of loss features is constant. When the peak is normalized against the loss features (yellow), the intensity at 618.7 eV is nearly doubled, which is supposed to prove the existence of triiodide as it has a similar BE as I^-.[4]

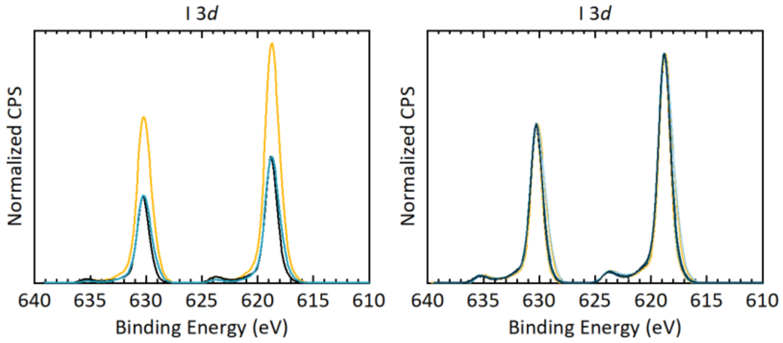

Figure S16 Left: background subtracted XPS I 3*d* spectra of Cs_2TeI_6 films after Peak IV. Blank sample is shown in black as the reference. Blue: Peak IV normalized against the highest intensity I^- peak. Yellow: Peak IV normalized against the energy loss peak. Right: background subtracted XPS I 3*d* spectra of all other samples.

Absorption spectra of the electrolytes were collected after CV measurements to understand the decomposition of perovskite during redox reactions. CsI, MAI, TeI_4 and elemental iodine were first sonicated in DCM electrolyte to identify the absorption peaks as in Figure S17 and all the absorbance are coming from iodine.

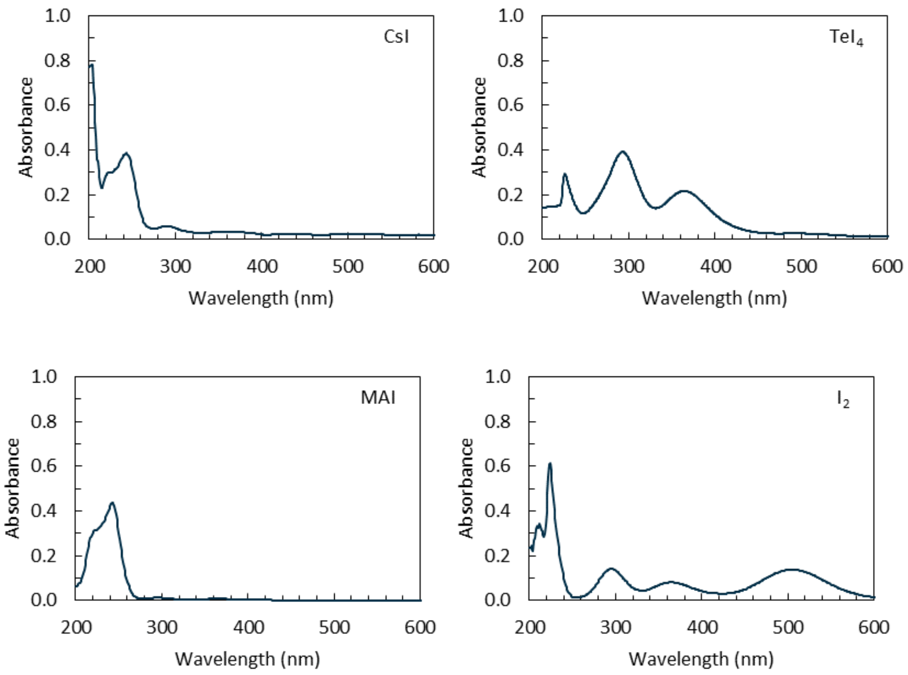

Figure S17 Normalized UV-vis absorption spectra of DCM electrolyte contains CsI, MAI, TeI_4 and iodine, respectively. Based on these results, the peaks at 220 nm and 244 nm are assigned to I^- ion, while the 294 nm and 365 nm peaks corresponding to I_3^-, iodine is assigned to the peak at 510 nm.[5]

Subsequent CV measurement of Cs_2TeI_6 was carried out within smaller potential range to understand the redox reactions. Results indicate that the oxidation of I^- does not happen between -0.3 to 0.6 V, and the absorption of electrolyte has a similar shape as CsI, which shows it contains mainly I^- ions that also remain on the surface.

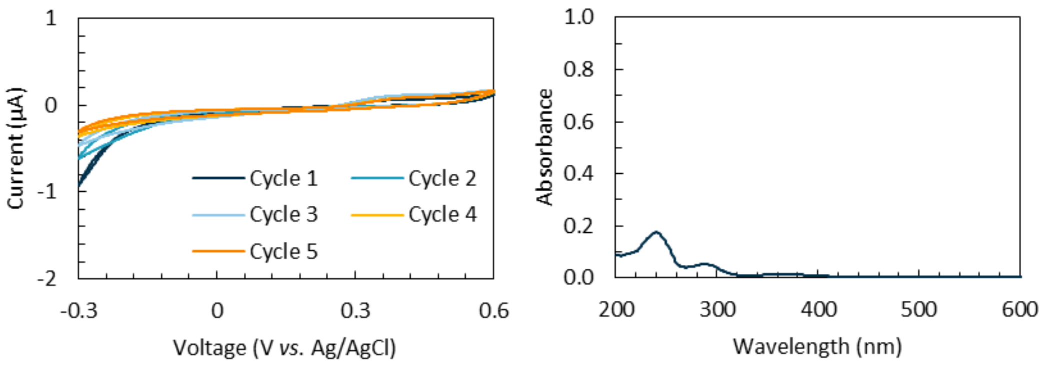

Figure S18 Left: subsequent CV measurements between -0.3 to 0.6 V (*vs.* Ag/AgCl) in DCM/$TBAPF_6$ at a scan rate of 5 mV/s. Right: the corresponding absorption spectra of electrolyte after measurement.

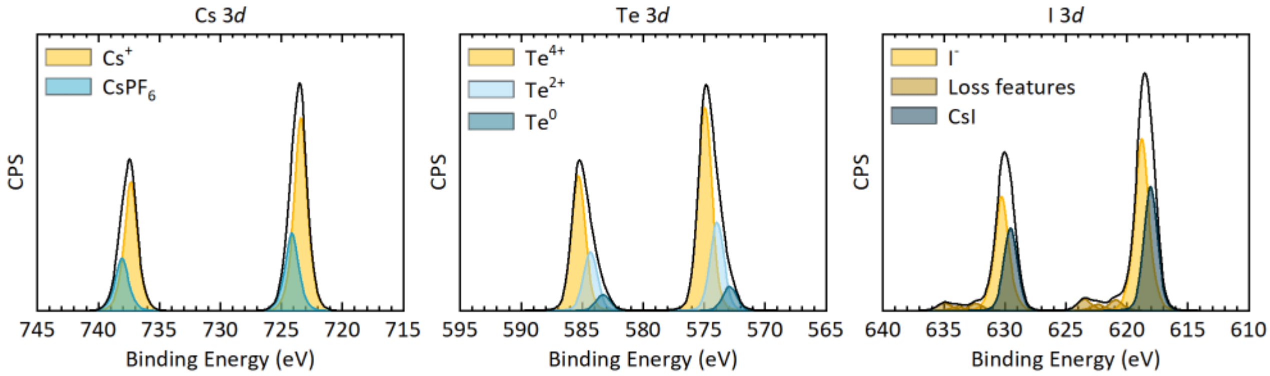

Figure S19 Background subtracted XPS core level spectra of Cs_2TeI_6 films after 5 CV cycles between -0.3 to 0.6 V (*vs.* Ag/AgCl).

The morphology of Cs_2TeI_6 films changes after redox reactions. Small cubes are formed on the surface of Cs_2TeI_6 after redox reactions as shown in Figure S20, which will eventually form a full

coverage on perovskite surface. EDS results show that F and P are enriched at this area, where Te and I are less than the surrounding area. This evidence of $CsPF_6$ formation on the surface after redox reactions is supported by XRD results as shown below. In addition, needle-like compound is observed only when reduction reaction happens, where Te mainly exists. Thus, this needle-like compound is believed to be tellurium metal formed during reduction reactions. The XRD pattern of Te metal is less obvious in Cs_2TeI_6 than MA_2TeI_6, which is believed to be the protective effect of $CsPF_6$.

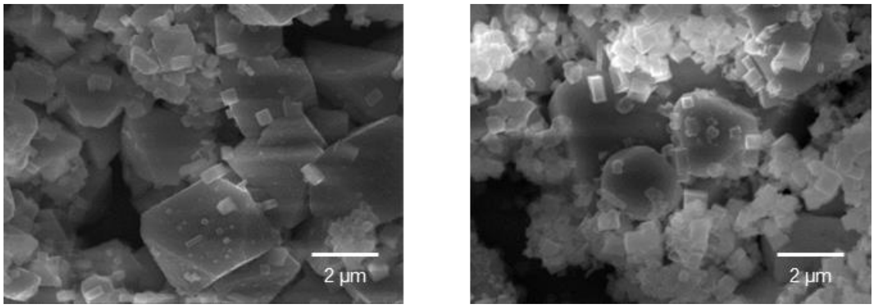

Figure S20 SEM images of the cubic formed on the surface of Cs_2TeI_6 film after redox reactions.

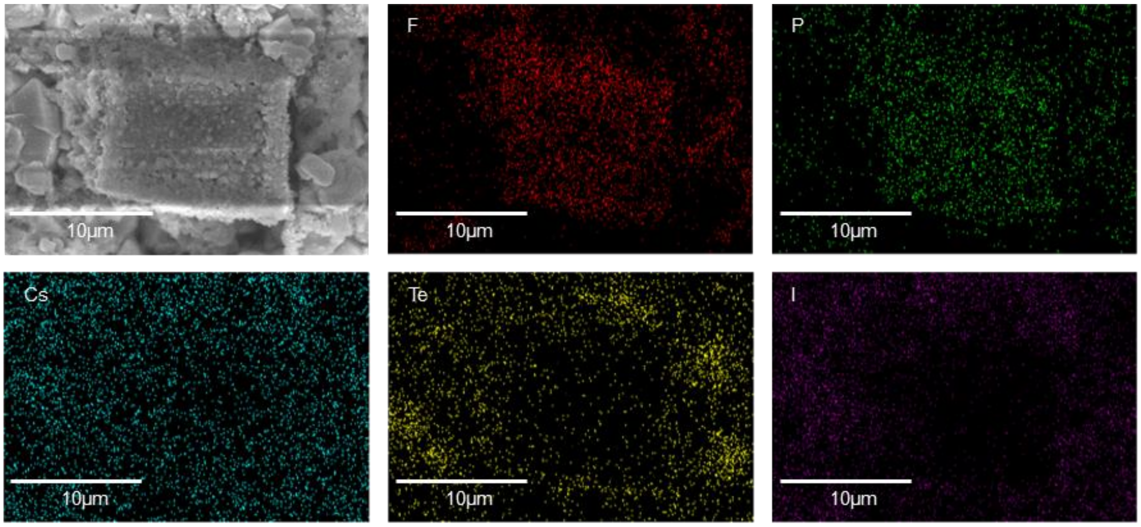

Figure S21 Elemental mapping of compact film formed on the surface of Cs_2TeI_6 film after redox reactions.

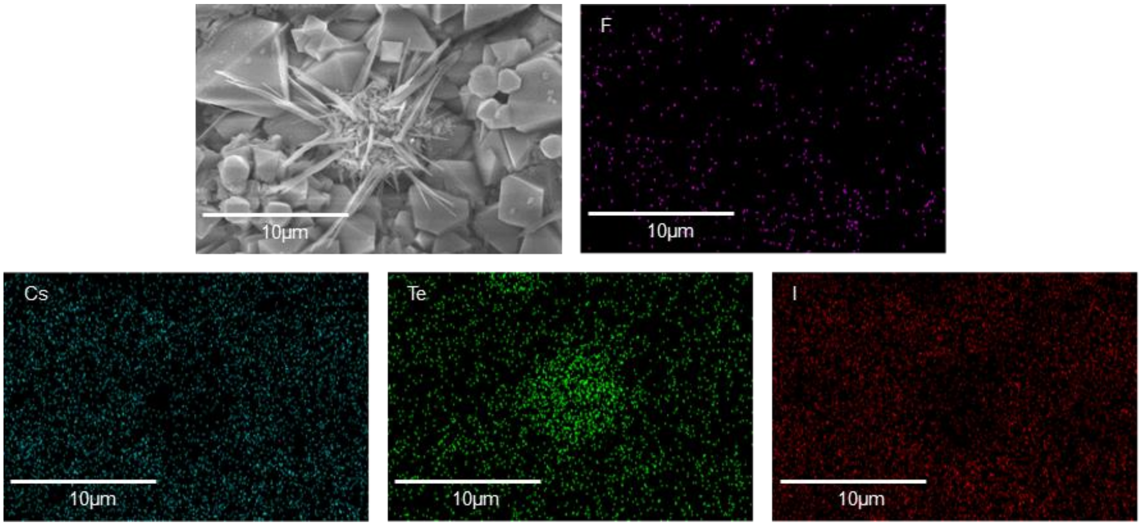

Figure S22 SEM images of needle-like compound formed at certain areas on Cs_2TeI_6 film after reduction reactions.

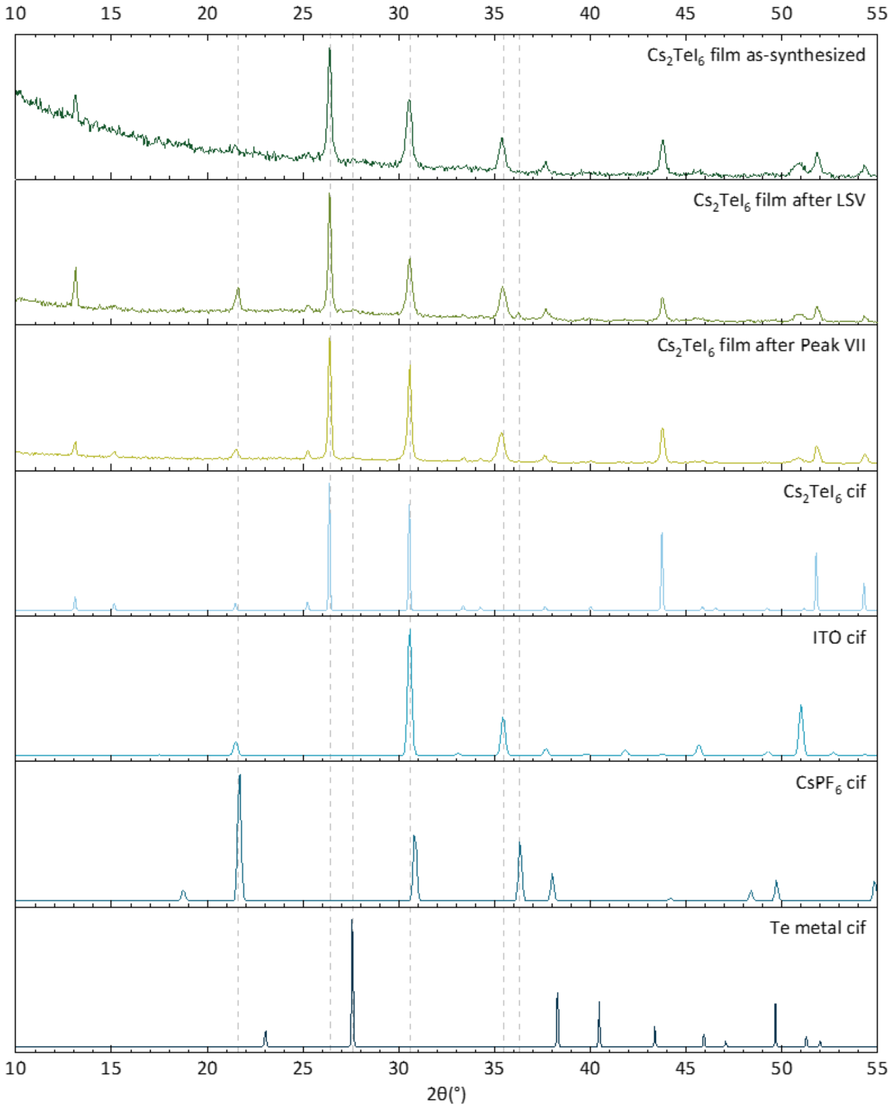

Figure S23 GIXRD pattern of Cs_2TeI_6 film before and after electrochemical reactions. The small peaks at 27.6° and 36.3° indicate the existence of tellurium metal and $CsPF_6$ formed during reduction reaction. The existence of Te metal is not as obvious as in MA_2TeI_6 as shown in Figure S30, which is believed to be a protective effect of $CsPF_6$.

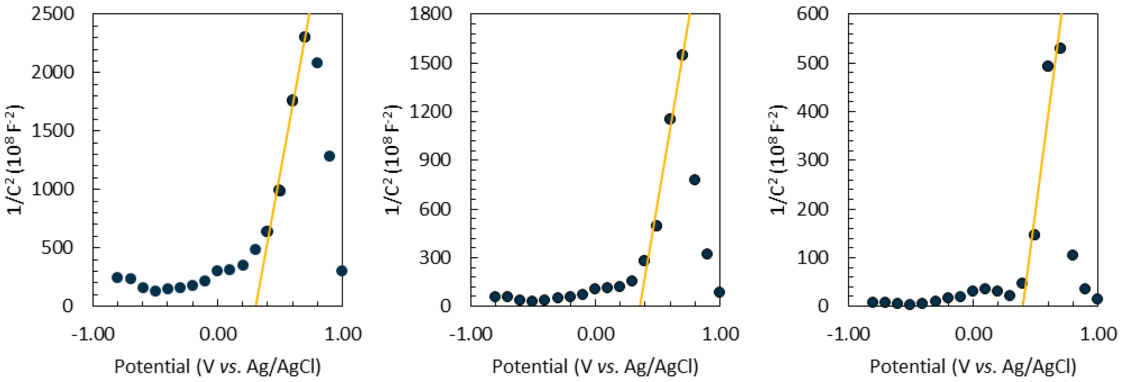

Figure S24 Mott-Schottky plot of Cs_2TeI_6 in DCM electrolyte at 2.254 Hz, 1.002 Hz and 0.3167 Hz respectively. The flat band potential is found to be around 0.38 V.

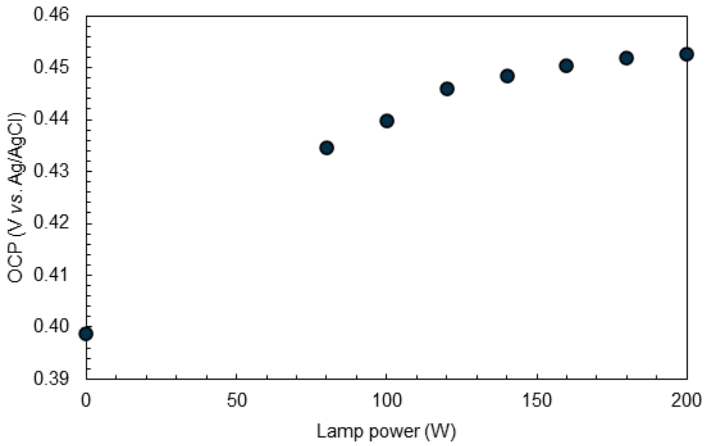

Figure S25 Open circuit potentials of Cs_2TeI_6 films that measured in dark and under different irradiance.

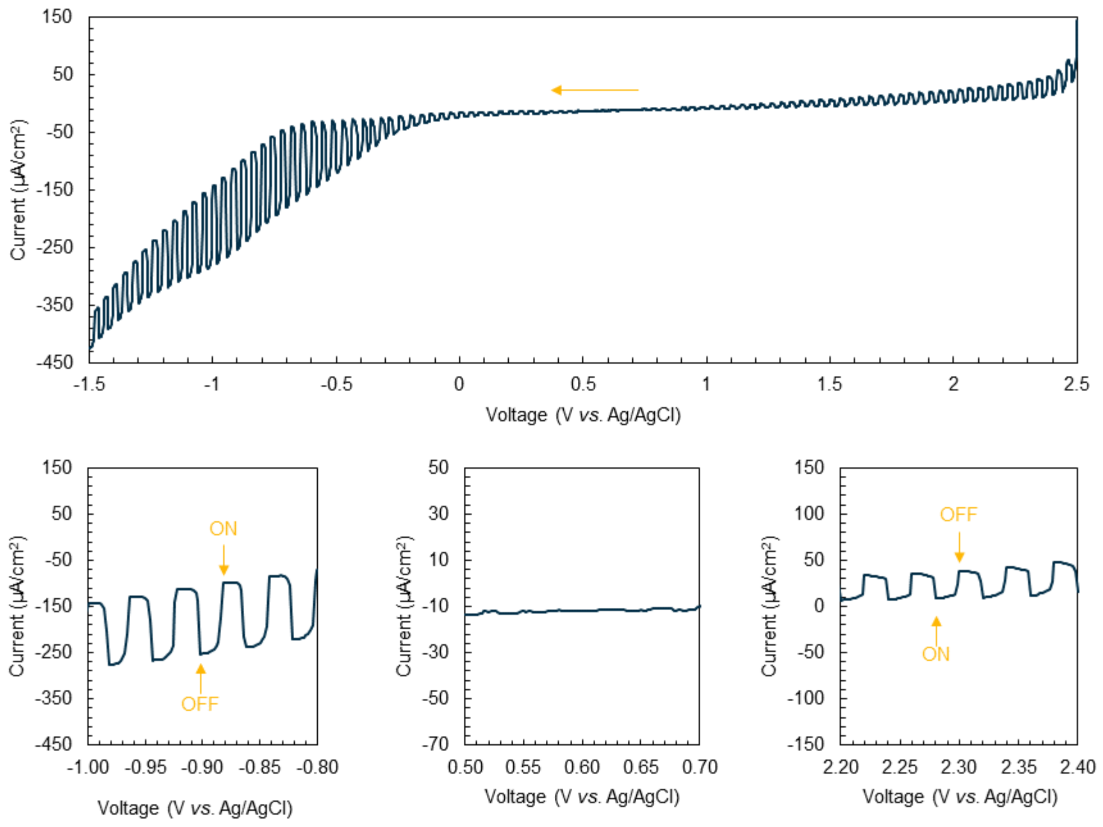

Figure S26 Chopped-light linear sweep voltammogram of Cs_2TeI_6 film which exhibits the photo-electrochemical photocurrent switching effect. Measurement was carried out in DCM/$TBAPF_6$ electrolyte at a scan rate of 2 mV/s. Arrow indicates the direction of scan.

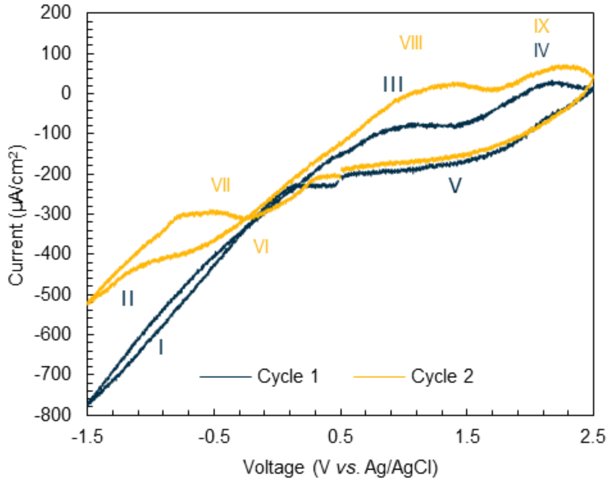

Figure S27 Cyclic voltammogram of MA_2TeI_6 film *vs*. Ag/AgCl, carried out in DCM/$TBAPF_6$ in dark at a scan rate of 5 mV/s.

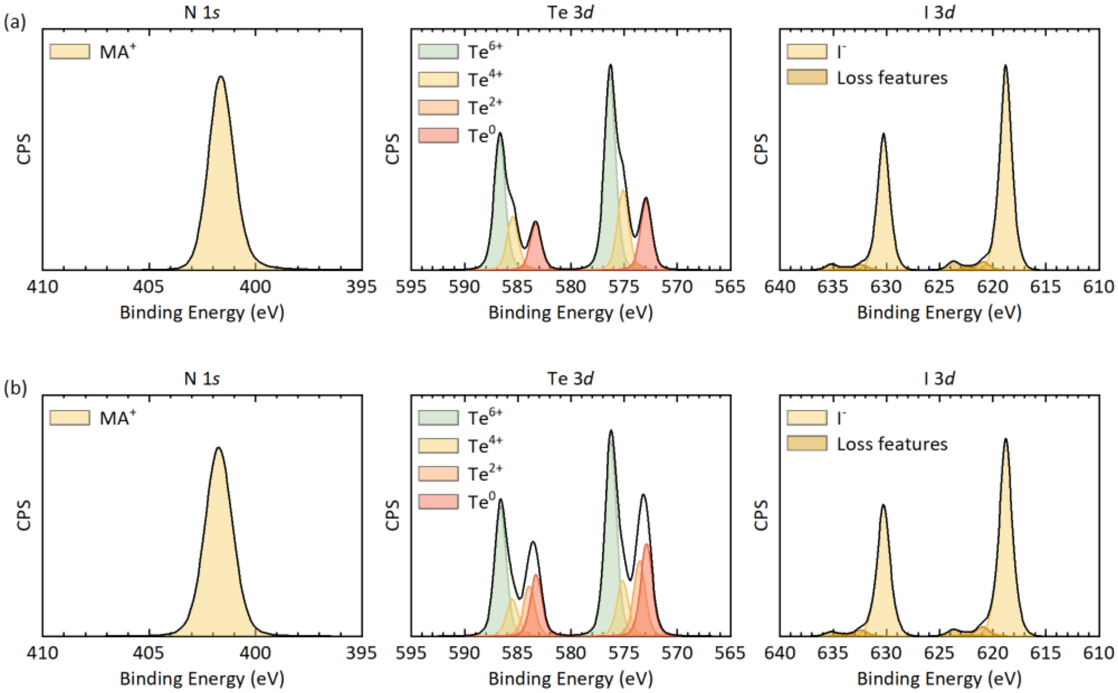

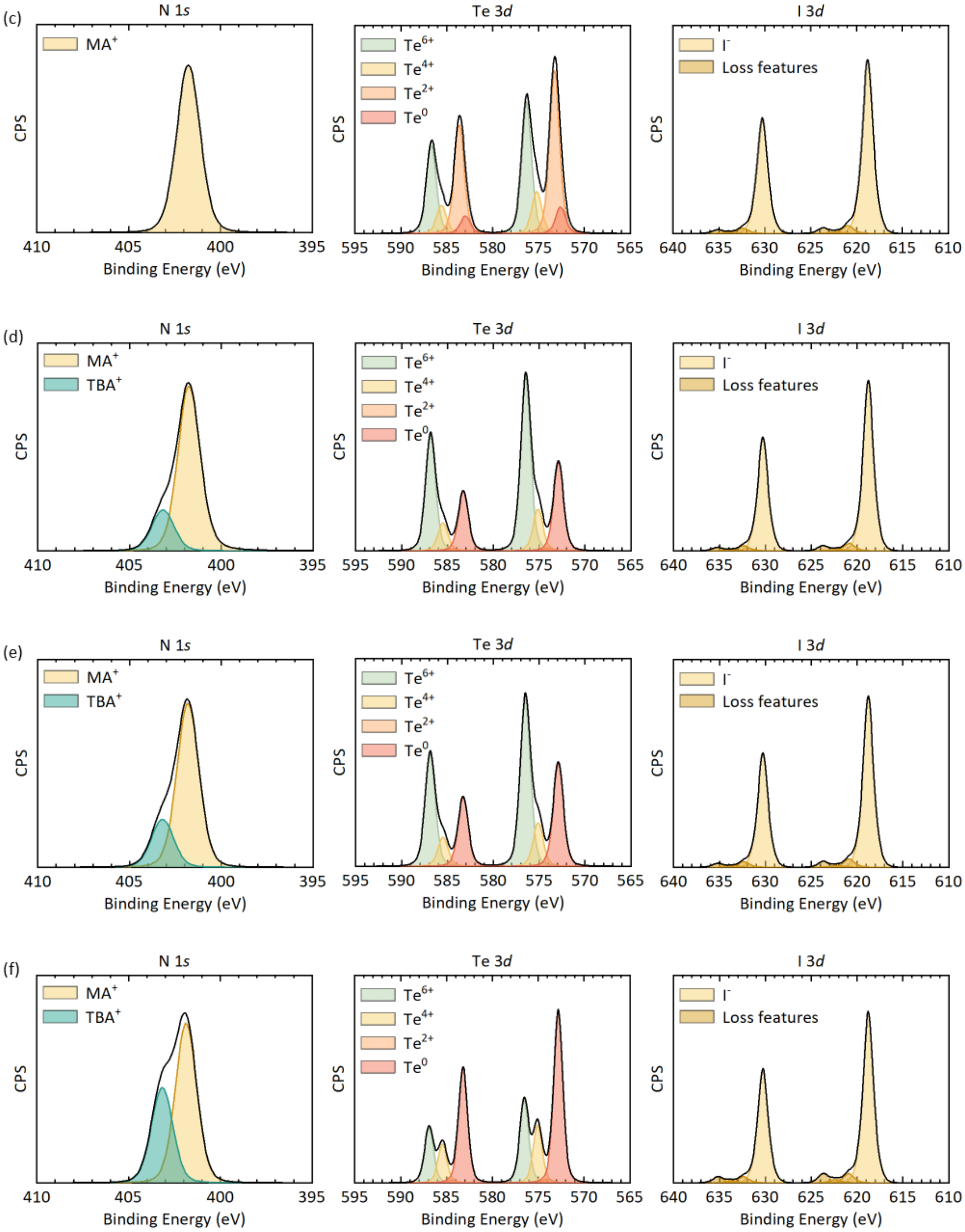

(c)
N 1s
MA+
CPS
410 405 400 395
Binding Energy (eV)
Te 3d
Te6+
Te4+
Te2+
Te0
CPS
595 590 585 580 575 570 565
Binding Energy (eV)
I 3d
I-
Loss features
CPS
640 635 630 625 620 615 610
Binding Energy (eV)
(d)
N 1s
MA+
TBA+
CPS
410 405 400 395
Binding Energy (eV)
Te 3d
Te6+
Te4+
Te2+
Te0
CPS
595 590 585 580 575 570 565
Binding Energy (eV)
I 3d
I-
Loss features
CPS
640 635 630 625 620 615 610
Binding Energy (eV)
(e)
N 1s
MA+
TBA+
CPS
410 405 400 395
Binding Energy (eV)
Te 3d
Te6+
Te4+
Te2+
Te0
CPS
595 590 585 580 575 570 565
Binding Energy (eV)
I 3d
I-
Loss features
CPS
640 635 630 625 620 615 610
Binding Energy (eV)
(f)
N 1s
MA+
TBA+
CPS
410 405 400 395
Binding Energy (eV)
Te 3d
Te6+
Te4+
Te2+
Te0
CPS
595 590 585 580 575 570 565
Binding Energy (eV)
I 3d
I-
Loss features
CPS
640 635 630 625 620 615 610
Binding Energy (eV)

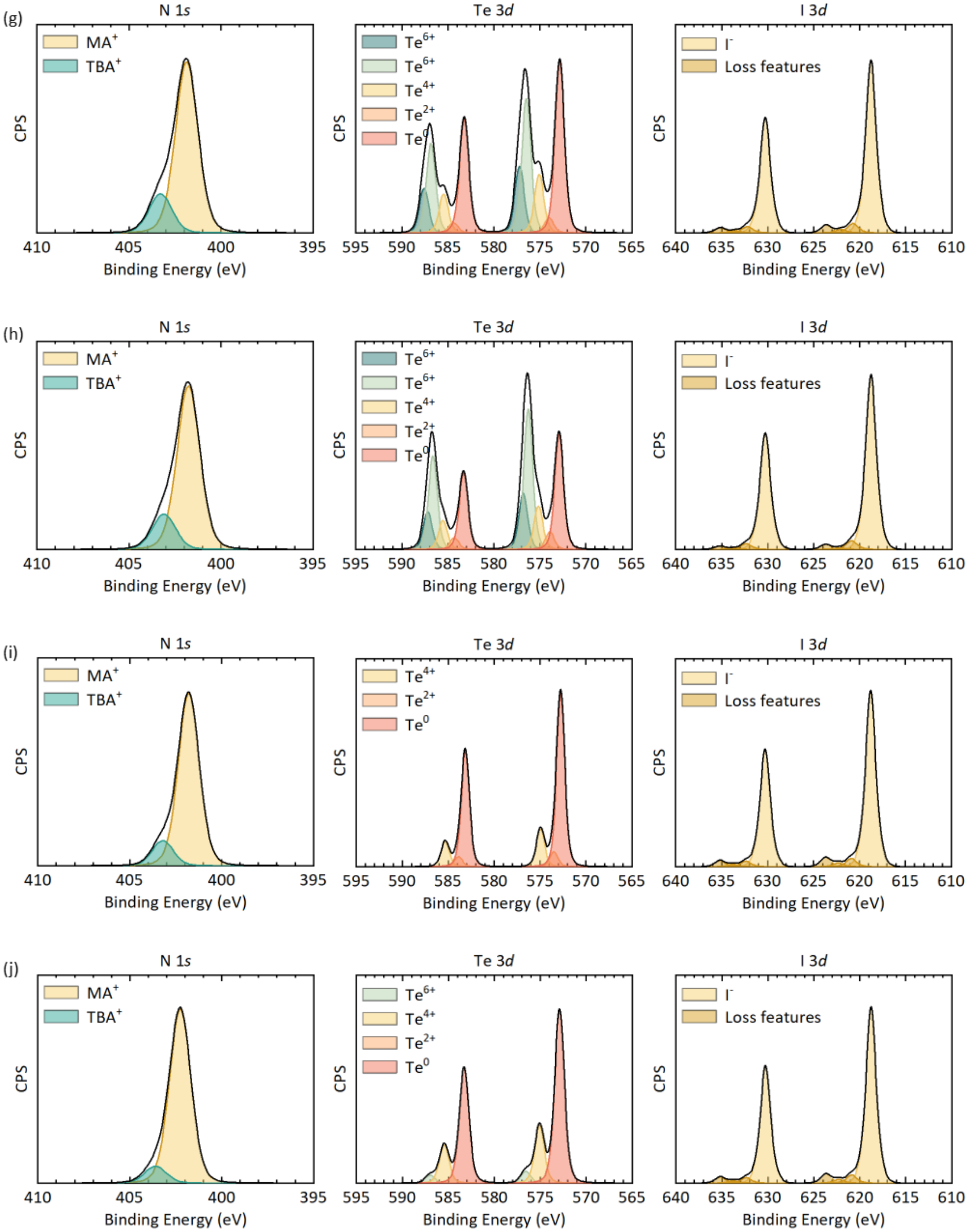
(g)
N 1s
MA+
TBA+
CPS
410 405 400 395
Binding Energy (eV)
Te 3d
Te6+
Te6+
Te4+
Te2+
Te0
595 590 585 580 575 570 565
I 3d
I-
Loss features
640 635 630 625 620 615 610
(h)
N 1s
MA+
TBA+
Te 3d
Te6+
Te6+
Te4+
Te2+
Te0
I 3d
I-
Loss features
(i)
N 1s
MA+
TBA+
Te 3d
Te4+
Te2+
Te0
I 3d
I-
Loss features
(j)
N 1s
MA+
TBA+
Te 3d
Te6+
Te4+
Te2+
Te0
I 3d
I-
Loss features

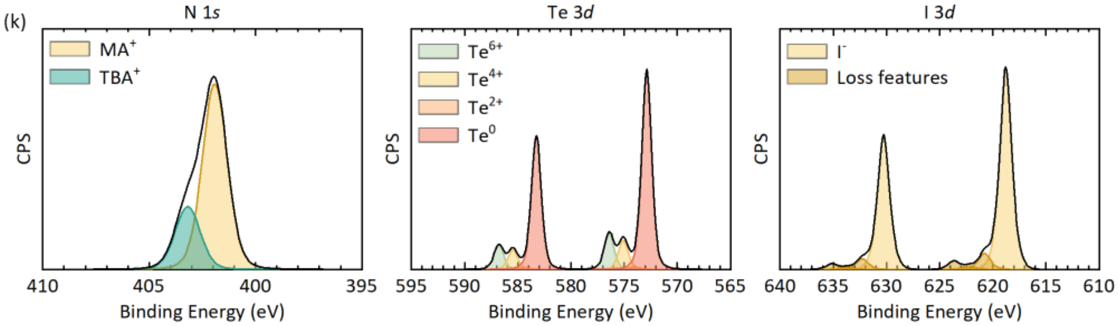

Figure S28 Background subtracted XPS core level spectra of MA_2TeI_6 films: (a) as-synthesized; after (b-j) peak I to peak IX in a partial CV scan and (k) after completing two CV cycles.

Table S3 Elemental ratio of MA_2TeI_6 film surface before and after electrochemistry reactions

	MA_2TeI_6			$TBAPF_6$		Unknown		
	N	Te	I	P	F	N	P	F
Blank	12.0%	19.7%	68.4%	-	-	-	-	-
Peak I	11.0%	24.9%	57.4%	0.9%	5.4%	-	0.4%	-
Peak II	10.7%	29.6%	55.0%	0.6%	3.6%	-	0.5%	-
Peak III	10.2%	17.8%	42.0%	4.0%	22.6%	2.5%	0.9%	-
Peak IV	9.2%	18.2%	38.5%	4.4%	25.8%	2.7%	1.2%	-
Peak V	6.8%	13.5%	35.6%	5.8%	32.4%	4.0%	1.9%	-
Peak VI	9.1%	18.9%	39.0%	3.8%	18.2%	2.1%	2.8%	6.0%
Peak VII	9.7%	18.7%	37.2%	4.7%	25.6%	2.2%	1.9%	-
Peak VIII	9.6%	14.7%	56.3%	2.4%	14.0%	1.5%	1.5%	-
Peak IX	10.3%	2.5%	14.1%	11.2%	55.6%	1.1%	0.5%	4.8%
After 2 CV cycles	8.3%	13.3%	29.8%	7.0%	34.3%	2.8%	1.4%	3.1%

The GIXRD data for MA_2TeI_6 films is shown below. The tellurium metal peak at 27.6° is more obvious compared to Cs_2TeI_6, which supports the protective effect of $CsPF_6$ on the surface. The peaks at 11.3°, 18.5° and 19.7° are believed to be three different compounds formed during experiments as their intensities vary across different samples, but they are hard to be identified.

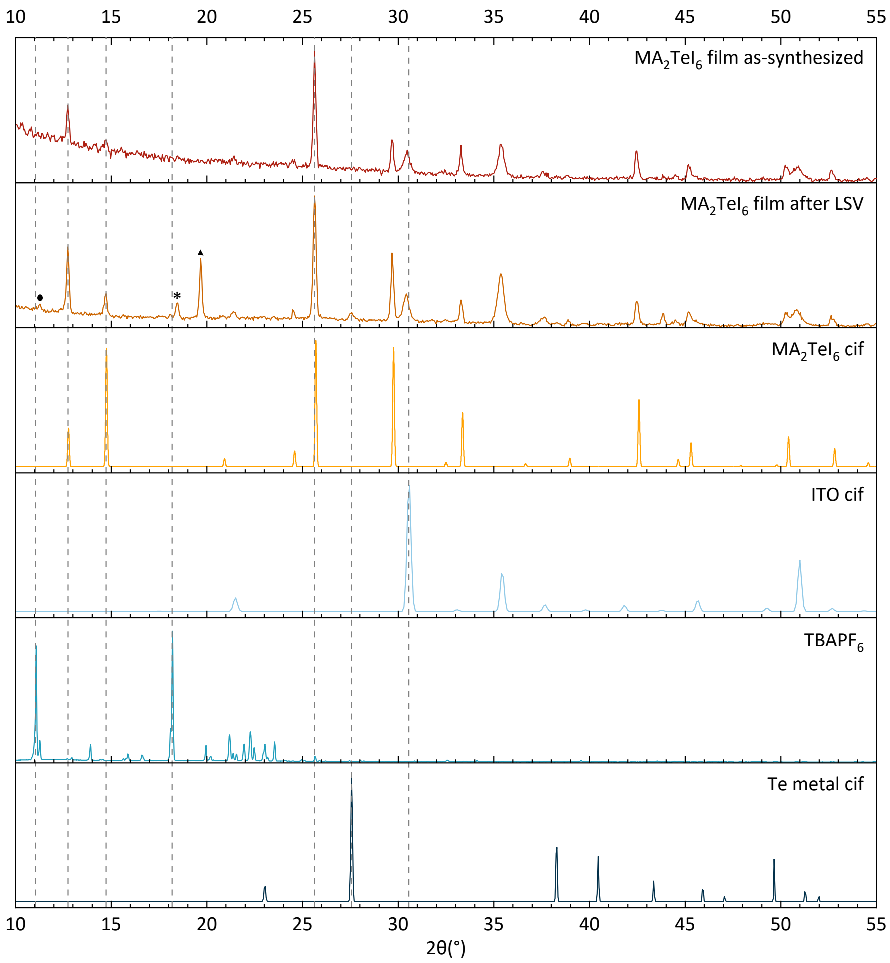

Figure S29 GIXRD pattern of MA_2TeI_6 film before and after electrochemical reactions. The small peaks at 27.6° indicates the existence of tellurium metal formed during reduction reaction, which is more obvious compared to Cs_2TeI_6.

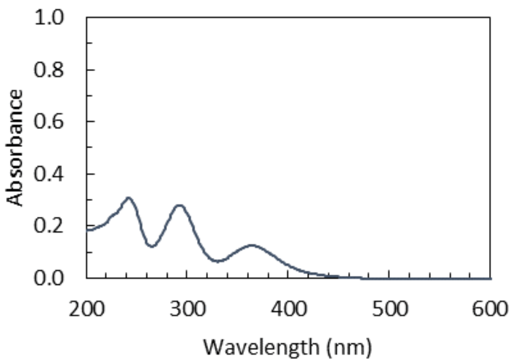

Figure S30 Normalized UV-vis absorption spectra of DCM electrolyte after the oxidation of MA_2TeI_6 film, which may contain TeI_4 and MAI.

The PEPS phenomenon is also present in MA_2TeI_6, both anodic and cathodic photocurrents are observed as the applied potential change from 2.5 to -1.5 V *vs.* Ag/AgCl. However, significant redox reactions take place during the chopped light measurement.

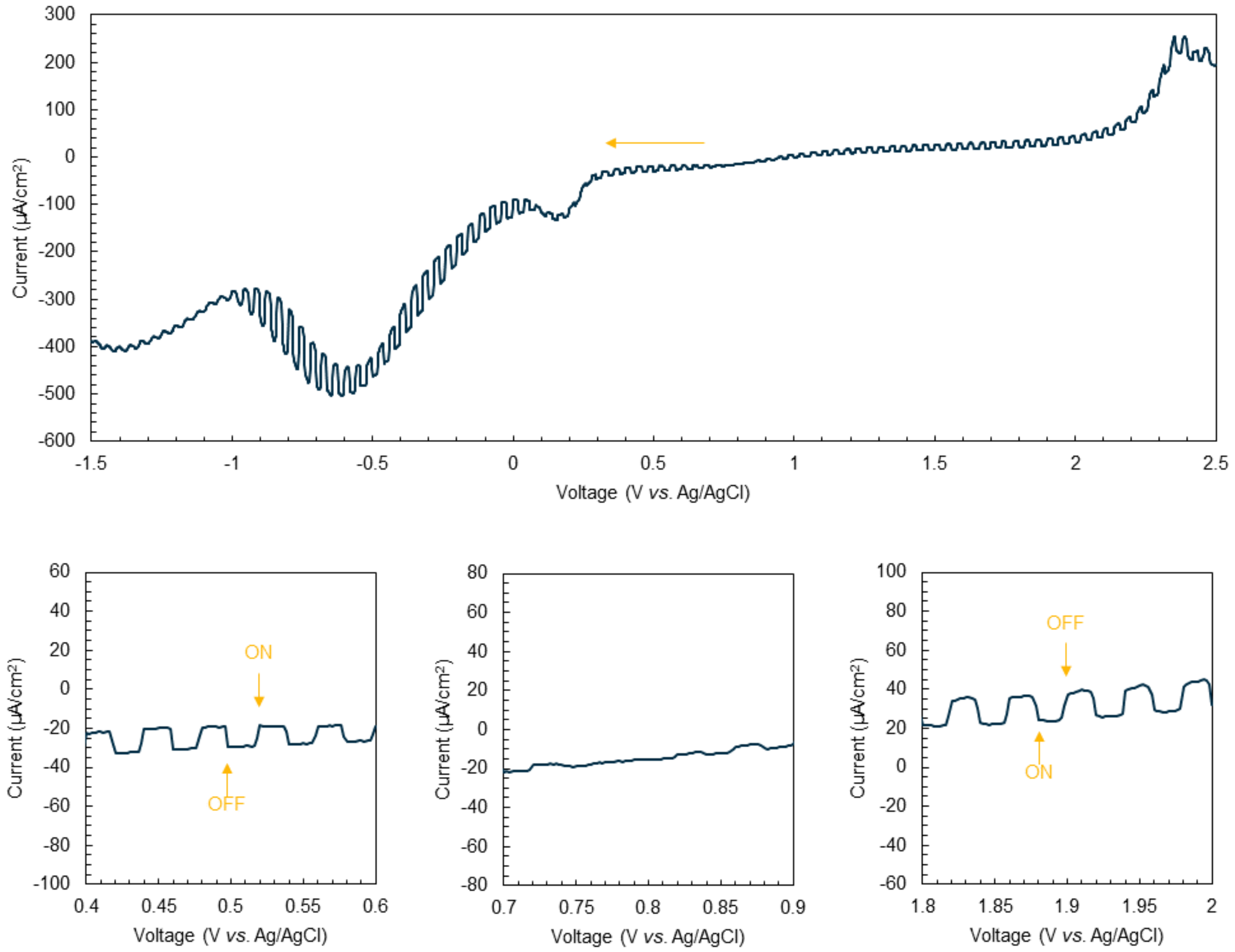

Figure S31 Chopped-light linear sweep voltammogram of MA_2TeI_6 film which exhibits the photo-electrochemical photocurrent switching effect. Measurement was carried out in DCM/$TBAPF_6$ electrolyte at a scan rate of 2 mV/s. Arrow indicates the direction of scan.

References

(1) Ju, M.; Dai, J.; Ma, L.; Zeng, X. C. Perovskite Chalcogenides with Optimal Bandgap and Desired Optical Absorption for Photovoltaic Devices. *Adv. Energy Mater.* **2017**, *7* (18), 1700216. https://doi.org/10.1002/aenm.201700216.

(2) Xiao, B.; Wang, F.; Xu, M.; Liu, X.; Sun, Q.; Zhang, B.-B.; Jie, W.; Sellin, P.; Xu, Y. Melt-Grown Large-Sized Cs2TeI6 Crystals for X-Ray Detection. *CrystEngComm* **2020**, *22* (31), 5130–5136. https://doi.org/10.1039/D0CE00868K.

(3) Moulder, J. F. *Handbook of X-Ray Photoelectron Spectroscopy*; Eden Prairie, 1995.

(4) Freeland, B. H.; Habeeb, J. J.; Tuck, D. G. Coordination Compounds of Indium. Part XXXIII. X-Ray Photoelectron Spectroscopy of Neutral and Anionic Indium Halide Species. *Can. J. Chem.* **1977**, *55* (9), 1527–1532. https://doi.org/10.1139/v77-213.

(5) Yang, W. S.; Park, B.-W.; Jung, E. H.; Jeon, N. J.; Kim, Y. C.; Lee, D. U.; Shin, S. S.; Seo, J.; Kim, E. K.; Noh, J. H.; Seok, S. Il. Iodide Management in Formamidinium-Lead-Halide–Based Perovskite Layers for Efficient Solar Cells. *Science (80-.).* **2017**, *356* (6345), 1376–1379. https://doi.org/10.1126/science.aan2301.
